# A sex-specific effect of M_4_ muscarinic cholinergic autoreceptor deletion on locomotor stimulation by cocaine and scopolamine

**DOI:** 10.3389/fnmol.2024.1451010

**Published:** 2024-12-16

**Authors:** Anna Berezovskaia, Morgan Thomsen, Anders Fink-Jensen, Gitta Wörtwein

**Affiliations:** ^1^Laboratory of Neuropsychiatry, Psychiatric Centre Copenhagen, Mental Health Services in the Capital Region of Denmark and University of Copenhagen, Copenhagen, Denmark; ^2^Copenhagen Center for Translational Research, Copenhagen University Hospital - Bispebjerg and Frederiksberg Hospital, Copenhagen, Denmark; ^3^Department of Neuroscience, Faculty of Health and Medical Sciences, University of Copenhagen, Copenhagen, Denmark; ^4^Department of Clinical Medicine, Faculty of Health and Medical Sciences, University of Copenhagen, Copenhagen, Denmark; ^5^Department of Public Health, Faculty of Health and Medical Sciences, University of Copenhagen, Copenhagen, Denmark

**Keywords:** acetylcholine, cholinergic receptor muscarinic 4, cocaine, ChAT(BAC)-Cre, autoreceptor

## Abstract

**Objective:**

Acetylcholine modulates the activity of the direct and indirect pathways within the striatum through interaction with muscarinic M_4_ and M_1_ receptors. M_4_ receptors are uniquely positioned to regulate plasticity within the direct pathway and play a substantial role in reward and addiction-related behaviors. However, the role of M_4_ receptors on cholinergic neurons has been less explored. This study aims to fill this gap by addressing the role of M_4_ receptors on cholinergic neurons in these behaviors.

**Methods:**

To investigate the significance of M_4_-dependent inhibitory signaling in cholinergic neurons we created mutant mice that lack M_4_ receptors on cholinergic neurons. Cholinergic neuron-specific depletion was confirmed using *in situ* hybridization. We aimed to untangle the possible contribution of M_4_ autoreceptors to the effects of the global M_4_ knockout by examining aspects of basal locomotion and dose-dependent reactivity to the psychostimulant and rewarding properties of cocaine, haloperidol-induced catalepsy, and examined both the anti-cataleptic and locomotion-inducing effects of the non-selective anticholinergic drug scopolamine.

**Results:**

Basal phenotype assessment revealed no developmental deficits in knockout mice. Cocaine stimulated locomotion in both genotypes, with no differences observed at lower doses. However, at the highest cocaine dose tested, male knockout mice displayed significantly less activity compared to wild type littermates (*p* = 0.0084). Behavioral sensitization to cocaine was similar between knockout and wild type mice. Conditioned place preference tests indicated no differences in the rewarding effects of cocaine between genotypes. In food-reinforced operant tasks knockout and wild type mice successfully acquired the tasks with comparable performance results. M_4_ receptor depletion did not affect haloperidol-induced catalepsy and scopolamine reversal of catalepsy but attenuated scopolamine-induced locomotion in females (*p* = 0.04). Our results show that M_4_ receptor depletion attenuated the locomotor response to high doses of cocaine in males and scopolamine in females, suggesting sex-specific regulation of cholinergic activity.

**Conclusion:**

Depletion of M_4_ receptors on cholinergic neurons does not significantly impact basal behavior or cocaine-induced hyperactivity but may modulate the response to high doses of cocaine in male mice and the response to scopolamine in female mice. Overall, our findings suggest that M_4_-dependent autoregulation plays a minor but delicate role in modulating specific behavioral responses to pharmacological challenges, possibly in a sex-dependent manner.

## 1 Introduction

Acetylcholine regulates numerous critical functions within the basal ganglia by activating nicotinic and muscarinic acetylcholine receptors (mAChRs). Growing evidence supports M_4_ as a crucial subtype of mAChR involved in modulating the dynamics of cholinergic and dopaminergic neurotransmission in striatum (Moran et al., 2019). This modulation is implicated in reward, learning, locomotion, and addiction-related behavior (Myslivecek, 2021).

The M_4_ receptor belongs to the metabotropic acetylcholine receptor family that is coupled via G_i/o_ and inhibits cAMP/PKA signaling (Caulfield, 1993). It is abundantly expressed in the striatum, being present in various cell types such as corticostriatal projection neurons, where it inhibits glutamate release (Pancani et al., 2014); cholinergic interneurons (ChIs), where it acts as an autoreceptor (Bernard et al., 1999; Yan and Surmeier, 1996); and finally, on direct pathway medium spiny projection neurons (dSPNs), where it opposes dopamine D_1_ receptor (D_1_R)-signaling (Foster et al., 2016; Nair et al., 2019).

Acetylcholine (ACh) and dopamine regulate the output of the striatum and facilitate circuit plasticity, both of which are fundamental for motor control and locomotion (Kreitzer and Malenka, 2008). Dopamine exerts its effects by stimulation of D_1_ receptors expressed on the dSPNs of the striatonigral pathway that leads to the disinhibition of thalamocortical circuits, promoting action execution and facilitating locomotion. In contrast, stimulation of D_2_ receptors (D_2_Rs) on spiny projection neurons from the indirect pathway suppresses motor activity and locomotion (Cui et al., 2013; Kravitz et al., 2010, 2012). Additionally, dopamine inhibits the activity of ChIs by acting on D_2_Rs (Chuhma et al., 2014; Wieland et al., 2014). On the other hand, ACh modulates the activity of both the direct and indirect pathways within the striatum predominantly through interaction with M_4_ and M_1_ receptors. It is suggested that M_4_ receptors are uniquely positioned to regulate plasticity within the direct pathway by acting on dSPNs, leading to a reduction in locomotion and opposing the initiation of movement (Foster et al., 2016; Gomeza et al., 1999; Nair et al., 2019).

Furthermore, M_4_ receptors play a substantial role in reward and addiction-related behaviors. Genetically engineered mice lacking functional M_4_ receptors (i.e., global M_4_ knockout mice) show increased cocaine and alcohol self-administration (De La Cour et al., 2015; Schmidt et al., 2011). Mice with cell type-specific knockout of M_4_Rs in D_1_R-dSPN, as well as global M_4_ knockout mice, exhibit heightened locomotor responses to psychostimulants and elevated dopamine release in the striatum following psychostimulant administration (Fink-Jensen et al., 2011; Jeon et al., 2010). Moreover, M_4_ Positive allosteric modulators (PAMs) attenuate hyperlocomotion induced by psychostimulants and reduce dopamine release in the striatum (Brady et al., 2008; Byun et al., 2014; Dall et al., 2017; Dencker et al., 2012).

While much attention has been paid to the role of M_4_R on D_1_R-expressing dSPN, the role of M_4_ receptors on cholinergic interneurons has been less explored. Comprising only 1-2% of the striatum cell population, cholinergic interneurons are the primary source of acetylcholine, providing widespread release of acetylcholine through dense and extensive axonal branching (Kawaguchi et al., 1995). Cholinergic interneurons are autonomous pacemakers (Bennett et al., 2000) and various neurotransmitters, neuromodulators, and synaptic inputs tightly regulate their activity (Poppi et al., 2021). Furthermore, ChIs hold promise as potential therapeutic targets for different neurological and psychiatric disorders such as Parkinson's disease, schizophrenia, and substance use disorder (Paul et al., 2022; Tanimura et al., 2018; Walker and Lawrence, 2020). Understanding the modulation of cholinergic cells in these disease states is critical for identifying pharmacological targets and developing effective treatments.

From *ex vivo* experiments, it is known that M_4_ autoreceptor activation reduces the opening voltage-gated calcium channels, which decreases the opening of small-conductance calcium-activated potassium channel after a neuronal spike, disrupting regular pacemaking activity (Ding et al., 2006; Yan and Surmeier, 1996). Additionally, M_4_ receptors activate a potassium conductance, possibly mediated by G protein-coupled inwardly rectifying potassium channels, which further slows down pacemaking (Calabresi et al., 1998). This modulation of neuronal activity ultimately reduces spiking at axon terminals, resulting in decreased acetylcholine release. Overall, it is believed that M_4_ receptor signaling functions as part of a negative feedback system to maintain optimal extracellular ACh levels (Calabresi et al., 1998; Ding et al., 2006). However, the functional significance of M_4_R modulation of ChIs has not been fully elucidated *in vivo*.

The overlapping expression pattern of M_4_ receptors in various neurons has made it difficult to determine its exact functions *in vivo*. Utilizing knockout animal models has proven helpful in addressing such challenges. We and others have previously demonstrated that mice with a global deletion of M_4_ exhibit hyperlocomotion, increased baseline dopamine levels, and heightened sensitivity to dopaminergic stimulants compared to wild types (Gomeza et al., 1999; Schmidt et al., 2011). Notably, depletion of the M_4_ receptor, specifically in D_1_-expressing medium spiny neurons, partially replicates this phenotype, suggesting its crucial role in modulating dopamine signaling in the striatum (Jeon et al., 2010). Here, to investigate the significance of M_4_-dependent inhibitory signaling in cholinergic neurons we created mutant mice that lack M_4_Rs specifically on cholinergic neurons. Firstly, to untangle the possible contribution of M_4_ autoreceptors to the effects of the global M_4_ knockout phenotype, we examined aspects of basal locomotion and dose-dependent reactivity to the psychostimulant and rewarding properties of cocaine. Secondly, we investigated how M_4_ receptors influence catalepsy induced by haloperidol and examined both the anti-cataleptic and locomotion-inducing effects of the non-selective anticholinergic drug scopolamine, given previous evidence that catalepsy is significantly reduced in global M_4_ knockout mice (Fink-Jensen et al., 2011). We also tested male and female mice to address potential sex-specific effects of M_4_ autoreceptor deletion, as behaviors mediated by the striatal dopamine system exhibit clear sex differences (Becker, 1999). Finally, a study by Klawonn et al. (2018) reported impairments in both classical and operant conditioning in male mice lacking M_4_ receptors on cholinergic neurons, created by utilizing the ChAT(IRES)-Cre line. In contrast, our current study employed the ChAT(BAC)-Cre driver line to delete M_4_Rs specifically from cholinergic neurons. Therefore we decided to attempt to replicate those previous findings by testing our ChAT(BAC)-Cre M_4_R male knockout mice in classically conditioned place preference and operant responding for food. We hypothesized that M_4_ autoreceptor deletion would partially recapitulate the effects of global M_4_ receptor knockout and that the effects on classical and operant behavior would be independent of the driver line used to create the knock-out.

This study provides insights into the role of muscarinic M_4_ receptors on cholinergic interneurons, highlighting how these receptors influence behaviors linked to reward, addiction, and movement in a sex-specific manner. By understanding M_4_ receptor function on cholinergic neurons, particularly in relation to dopamine signaling and behavioral regulation, these findings could inform therapeutic strategies for neuropsychiatric conditions such as schizophrenia, substance use disorders, Parkinson's disease, and other conditions with sex-dependent characteristics.

## 2 Materials and methods

### 2.1 Animals

Male and female mice (8–16 weeks) inbred on C57BL/6J were used for all experiments. The mice were maintained on a reversed 12-h light/dark cycle (light on at 7 p.m.) with *ad libitum* access to food and water. The mice were group housed in individually ventilated cages enriched with cardboard housing, tunnels, wooden chewing blocks, and nesting material. The mice were always habituated to the testing room for a minimum of 45 min before every experiment. All procedures were ethically approved by the Animal Experiments Inspectorate under the Danish Ministry of Food, Agriculture, and Fisheries, according to the EU directive 2010/63/EU.

Muscarinic M_4_-ChAT-Cre^+^ knockout animals were generously provided by Professor Jürgen Wess (Laboratory of Bioorganic Chemistry, National Institute of Diabetes and Digestive and Kidney Diseases, National Institutes of Health, USA) and bred in our facility. To achieve the conditional knockout of M_4_ mAChRs on cholinergic neurons, the previously described M_4_ mAChR flox strain was used (Jeon et al., 2010). Crossbreeding with ChAT(BAC)-Cre mice (GM60Gsat/Mmucd, RRID: MMRRC_030869) obtained from the GENSAT project (Gong et al., 2007) resulted in the generation of M_4_-ChAT-Cre+ mice (later referred to as KO mice), wherein M_4_ mAChR was selectively lacking in cholinergic neurons. Control littermates, M_4_-ChAT-Cre^−^ (referred to as WT), were homozygous for the M_4_ flox allele but lacked the Cre transgene. Genotyping was performed through PCR analysis of mouse ear DNA using specific primers for ChAT-Cre (forward primer 5′-GGTCTCCTTGTGGAGTGGGAGT-3′, reverse 5′-CGGCAAACGGACAGAAGCATT-3′) and M_4_ flox (forward primer 5′- TGCAGATGTAGCTCAGCTCAGCGGTAC-3′, reverse5′-TGAAGGTTGTAGACAAAGCTATACACATGGC-3′).

### 2.2 Validation of cholinergic neuron-specific M_4_ mAChR depletion

#### 2.2.1 In situ hybridization

Brains were dissected, quickly frozen on dry ice, and stored at −80°C until being sectioned on a cryostat (CM3050 S, Leica Microsystems, Wetzlar, Germany) at 10 μm. The RNAscope fluorescent *in situ* hybridization process was conducted as specified in the manufacturer's protocol (Advanced Cell Diagnostics, California, USA). Briefly, the slides were fixed for 15 min in an ice-cold 4% PFA buffered solution. Subsequently, slides were dehydrated through a series of ethanol solutions of increasing concentrations and were left to air dry. The slides were then treated with Protease-IV for 30 min at RT, followed by a 2-h incubation at 40°C with Mm-Chrm4-C2 (catalog #410581, Advanced Cell Diagnostics) and Mm-Chat-C1 (catalog #408731, Advanced Cell Diagnostics) probes. After probe incubation, four amplification steps were conducted with intervening washing steps. Finally, the slides were counterstained with DAPI and coverslipped with an aqueous mounting medium (catalog #ab104135; Abcam, Massachusetts, USA). Slides were imaged using the Zeiss Axioscan 7 (Carl Zeiss AG, Oberkochen, Germany), an automated slide-scanning system. The dorsal and ventral striatum from three sections for each mouse were captured, and the labeled cells were quantified using QuPath (Bankhead et al., 2017). Cells were considered positive by the observer if there were more than 5 particles around the nucleus.

### 2.3 Power analysis

Based on the data published by Klawonn et al. (2018) power analysis was conducted to determine the required sample size to detect a significant difference in activity levels between knockout and wild type animals aiming at a power of 0.80. The analysis was based on two-sample *t*-test for independent groups. This suggested a sample size of n = 8 per group.

### 2.4 SHIRPA behavioral test

The SHIRPA primary screen procedure was employed to evaluate the behavioral and physical characteristics of drug and experimentally naive mice and their wild type littermates, encompassing an assessment of basic reflexes and sensorimotor functions (Lalonde et al., 2021). The assessment of each animal started with a 5-min observation of undisturbed behavior in a cylindrical viewing jar. Mice were then transferred to a plexiglass cage for motor behavior examination. Subsequently, visual acuity, grip strength, body tone, and reflexes were recorded. Autonomic responses, including changes in skin color and heart rate, were documented, and body length was measured. Limb tone, salivation, and provoked biting responses were also monitored. The primary screen was finalized with measurements of the righting reflex, contact-righting reflex, negative geotaxis, and a wire maneuver to assess the animals' hind leg gripping ability when hung by the forelimbs. Any unusual behavior, fear, agitation, hostility, or human-audible vocalization episodes were documented during the testing procedure. The testing area was cleaned and dried after each mouse. One day later, locomotor activity was recorded using Ethovision^®^XT (version 16-17; Noldus, Wageningen, Netherlands) for 1 h in open field boxes.

### 2.5 Drugs

Cocaine hydrochloride was obtained from the Copenhagen University Hospital Pharmacy (Copenhagen, Denmark) and dissolved in 0.9% saline. Scopolamine hydrobromide was purchased from Sigma-Aldrich and Haloperidol (Serenase, 5 mg/ml, Janssen-Cilag, Denmark) from Janssen-Cilag. All drugs were dissolved in saline and administered intraperitoneally in a total volume of 10 μl/g body weight.

### 2.6 Locomotor activity

Locomotor activity was evaluated in an open field (40 × 40 × 80 cm) situated in a dimly lit room (16 lux) with an overhead camera. The Ethovision^®^XT tracking system (Noldus) was used to analyze the distance moved. Cocaine doses of 5, 10, 15, and 40 mg/kg were administered in the experiment. Wildtype and knockout males and females underwent a 30-min habituation period in the open field before intraperitoneal cocaine injection, with locomotor activity monitored for the subsequent 60 min. Additionally, the male cohort of mice injected with 40 mg/kg was manually scored for cocaine-induced stereotypy. For the detailed protocol see Supplementary material 1.2.

Scopolamine is a competitive, non subtype-selective antagonist of mAChRs which causes dose-dependent alterations in locomotor behavior (Itzhak and Martin, 2000; Thomsen, 2014). To evaluate the impact of scopolamine on locomotion, both male and female subjects were initially acclimated to the activity chambers (MED-OFA-510, Med Associates, Vermont, USA) for a period of 60 min. Following this habituation phase, the mice received an injection of 1 mg/kg scopolamine, and their locomotor activity was recorded for an additional 60 min. Two weeks later, the same cohort underwent a similar procedure except that the dose of scopolamine was increased to 3 mg/kg.

### 2.7 Cocaine sensitization

To induce behavioral sensitization, we utilized a combination of repeated cocaine injections and daily 60-min exposures of the naïve male mice to activity chambers (MED-OFA-510, Med Associates) as an environment distinct from the home cage. The experiment commenced with a baseline day (day 0), where all mice received saline injections. Subsequently, knockout and wild type mice were stratified into two groups to ensure an equal distribution of both high and low locomotor activity phenotypes across the saline and cocaine treatment groups. Over the course of six consecutive days (days 1 to 6), these groups were subjected to daily injections paired with exposure to the activity chambers, with one group receiving cocaine at a dosage of 15 mg/kg and the other receiving saline. Following a 2-week test-free period, all mice were administered cocaine, and locomotor activity was evaluated in the activity chambers on day 20. On the subsequent day (day 21), all mice were injected with saline and again exposed to the chambers to assess the extent to which the observed hyperlocomotion was attributable to contextual conditioning as opposed to the direct effects of cocaine.

### 2.8 Conditioned place preference (CPP)

The rewarding effects of cocaine were evaluated with the conditioned place preference paradigm. In this widely used test, animals are classically conditioned to associate a particular environment or context with the drug's effects. The Open field activity boxes (MED-OFA-510, Med Associates) with a beam-break movement detection system were transformed into a two-compartment arena for the CPP experiment. A red plastic partition with an opening was used to separate the two compartments. One compartment had a metal grid floor, and the other one had a dark gray Lego^®^ plate floor. There was no systematic preference for either of the floors. Each arena was individually placed in a sound-attenuating cubicle equipped with a light (40 lux). Only males were used in the experiment. During the 15-min pretest, animals were allowed to move freely between the two compartments, and the time spent in each compartment was measured. Based on the data from the pretest, cocaine injection was paired with the least preferred compartment for an individual animal. During conditioning, the opening between the two compartments was closed, and mice were injected with saline in one of the compartments. Approximately 4 h later, animals were injected with 15 mg/kg cocaine in the opposite compartment. Acquisition lasted five consecutive days, 24 h after acquisition animals underwent a 15-min post-test assessment with an opened partition. During the extinction phase, which lasted six consecutive days, the animals underwent 1 h sessions where they received saline injections in both compartments twice daily. Twenty-four hours after the last extinction session, mice went through an extinction test. For reinstatement, animals were injected with 5 mg/kg cocaine immediately before the test. The individual conditioning preference score was determined by subtracting the time spent in the cocaine-paired compartment during the pretest from the time spent in the same compartment during the posttest. Similarly, the reinstatement score was calculated by subtracting the time spent in the cocaine-associated compartment during the last day of extinction test from the time spent in the same compartment during the reinstatement test (Klawonn et al., 2018).

### 2.9 Food self-administration

A cohort of male mice underwent food self-administration training following a protocol detailed in a previously published work (Thomsen et al., 2009). Modular operant-conditioning chambers (ENV-307A, Med Associates) were employed, equipped with a house light, two nose-poke holes each containing a yellow cue light, and a steel dish into which liquid food reinforcers were delivered from a syringe pump. Throughout the experiment, mice were maintained under mild food restriction conditions. Before the study began, mice were introduced to the reinforcer in the home cage, the reinforcer being vanilla-flavored Nutridrink (Nutricia, Utrecht, Netherlands). Subsequently, mice underwent daily 2-h sessions under a fixed ratio 1 schedule of reinforcement (FR 1) with a timeout of 20 s, with one active and one inactive nose-poke hole. Training persisted for a minimum of 5 days, during which mice had to meet the criteria: earning a minimum of 20 reinforcers in two consecutive sessions, displaying no more than 20% variation in responses, and exhibiting a minimum of 70% responses in the active hole in both sessions. Following FR 1 training, extinction sessions with water were conducted for a minimum of two sessions, continuing until the response rate dropped to < 80% of its initial level. Various liquid food dilutions (water, 3, 10, 32, 100%) were then introduced based on a Latin square design. Mice meeting criteria under the FR 1 schedule proceeded to progressive ratio (PR) sessions, in which response requirement (ratio) incremented by 0.115 log units after each reinforcer delivery. PR sessions terminated when a 60-min limited hold period elapsed with no reinforcers being earned, or after 6 h, whichever occurred first. The breaking point was defined as the value associated with the final completed ratio (the number of earned reinforcers). After achieving a consistent response (two consecutive sessions with breaking points exceeding 5 and displaying < 20% variation), water was substituted until the response rate diminished to < 50% of the baseline. Similar to the FR schedule, concentration-effect curves were determined, with each dose tested for at least two consecutive sessions. We limited the operant learning task to male mice to reduce variability and establish a baseline for M_4_ receptor function on cholinergic neurons in an operant context, acknowledging that females' increased sensitivity to scopolamine could introduce additional complexity in interpreting results.

### 2.10 Haloperidol-induced catalepsy

The administration of traditional antipsychotic medications like haloperidol is often accompanied by significant motor side effects, which are partially explained by changes in dopaminergic and cholinergic neurotransmission in the striatum. The bar test, a commonly utilized animal model, assesses these motor side effects (Sanberg et al., 1988). Scopolamine, a non-specific muscarinic antagonist was shown to reverse these effects (Jeon et al., 2010). For the experiment, knockout and wild type mice previously exposed to cocaine were reused after a two-week test-free period. Mice were administered haloperidol intraperitoneally (0.3 and 1.0 mg/kg). They were kept in their home cage until catalepsy testing at 30, 60, and 90 min. Testing involved placing the mice with front paws on a steel bar (15 cm long, 0.5 mm diameter, 5.5 cm high) and timing their stay in this position with a 120-s cut-off. Mice failing to maintain position were retested twice, recording zero seconds if unsuccessful. To investigate the reversal of cataleptic effects, all mice were given scopolamine (0.5 or 1 mg/kg) immediately after the final test at 90 min, and 30 min later, the mice were assessed for any residual cataleptic reactions.

### 2.11 Statistical analysis

Data are presented as mean ± SEM if not stated otherwise. Results were considered statistically significant if *p* < 0.05. In all experiments, the age distribution was balanced across groups. GraphPad Prism 9 (GraphPad Software Inc., San Diego, CA, USA) was used to perform statistical analyses. ANOVA with Geisser-Greenhouse correction as appropriate followed by Šídák's multiple comparison *post hoc* test or *t*-test was used as appropriate. The log-rank test was used to analyze the number of sessions required for KO and WT mice to reach criteria in an operant learning task.

## 3 Results

### 3.1 Validation of cholinergic neuron specific muscarinic M_4_ mAChRs depletion in knockout mice

**Figure 1:**
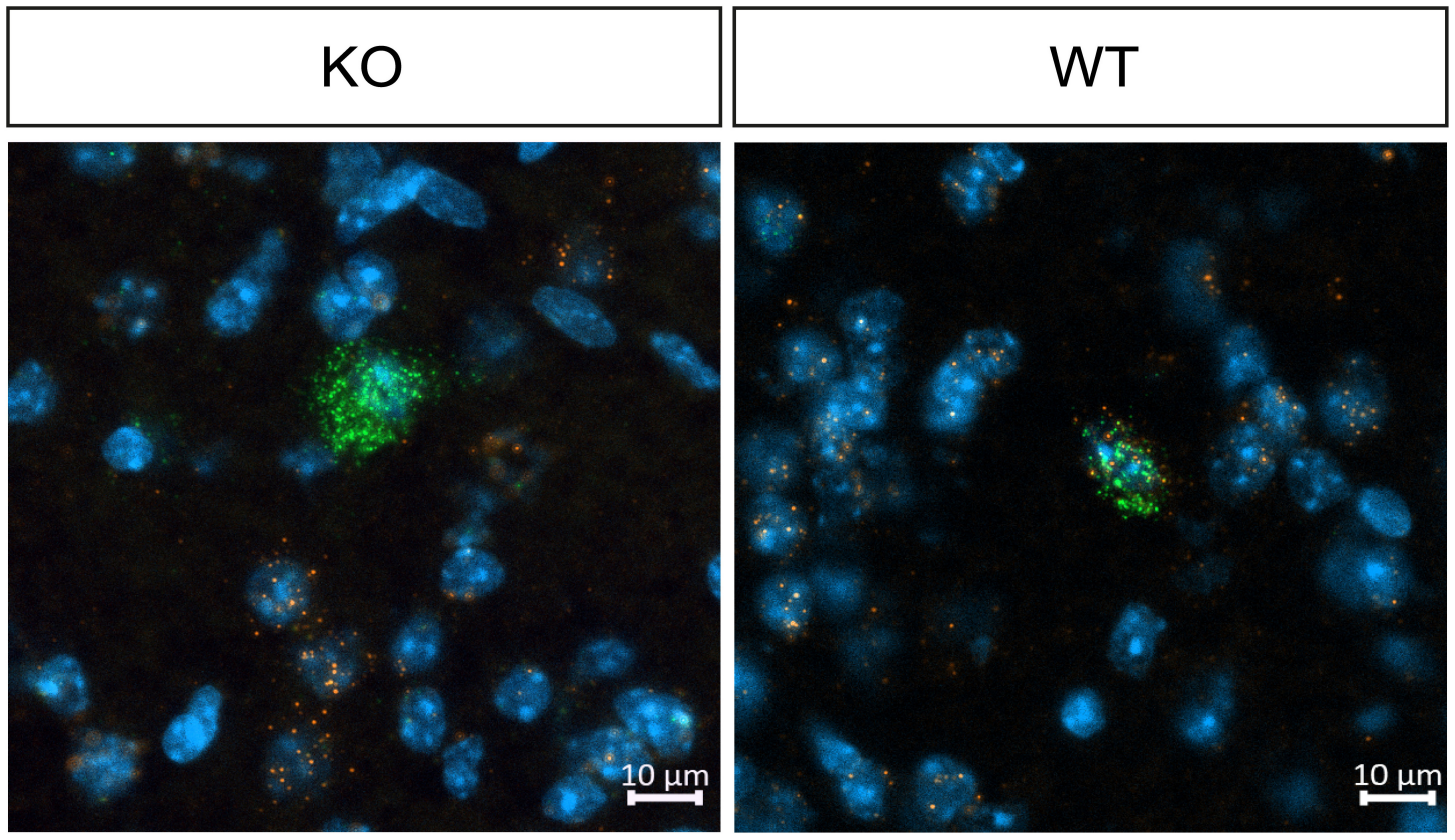
Representative images of fluorescent *in situ* hybridization in WT and KO mice, demonstrating effective M_4_ receptor knockout in cholinergic neurons. In wild type mice, Chrm4 mRNA is present in all ChAT-expressing cells, whereas knockout mice display minimal co-expression of Chrm4 and Chat mRNA, confirming successful depletion of M4 mAChR on cholinergic neurons. *n* = 2 per group.

To confirm the cholinergic neuron-specific depletion of the M_4_ receptor in the knockout mice, we conducted *in situ* hybridization to examine the expression of striatal Chrm4 mRNA. In wild type mice, Chrm4 mRNA was present in all ChAT-expressing cells. In contrast, the knockout mice displayed minimal co-expression of Chrm4 and Chat mRNA, with only about 2% of cells being double-positive (see Figure 1 and Table 1). This limited co-expression might be attributed to the proximity of D1-MSN and cholinergic interneurons in the striatum. As another way of validating cell-type specific knockout, a separate group of mice was unilaterally injected with a Cre-dependent adeno-associated virus in the dorsal striatum. Analysis of the eYFP signal supports that Cre-recombinase expression was exclusive to the knockout mice with no detectable signal in wild type animals (Supplementary Figure 1). Thus, together, fluorescent *in situ* hybridization and Cre-recombinase expression pattern confirm the effectivity and specificity of the knockout.

**Table 1:** Validation of M_4_ mAChR knockout.

	**WT**	**KO**
Chat^+^ Chrm4^+^	122 (100%)	3 (2%)
Chat^+^ Chrm4^−^	0 (0%)	144 (98%)

Number of cells with the percentage in brackets. Chrm4 (orange) and Chat (green) with DAPI (blue) for nuclei. Fluorescent *in situ* hybridization confirmed the efficacy of the conditional knockout of M_4_ mAChR in cholinergic cells.

### 3.2 Basal phenotype

Initially, naïve males and females from both genotypes were subjected to SHIRPA primary screening (Lalonde et al., 2021). The procedure showed that KO mice have normal weight, size, reflexes, and other physiological parameters compared to WT littermates, as detailed in Supplementary Table 1. Additionally, as part of the health evaluation, basal locomotor activity in an open field was measured for 1 h, and no differences were found (*p* = 0.58) (Supplementary Figure 2).

### 3.3 Cocaine-induced hyperlocomotion

**Figure 2:**
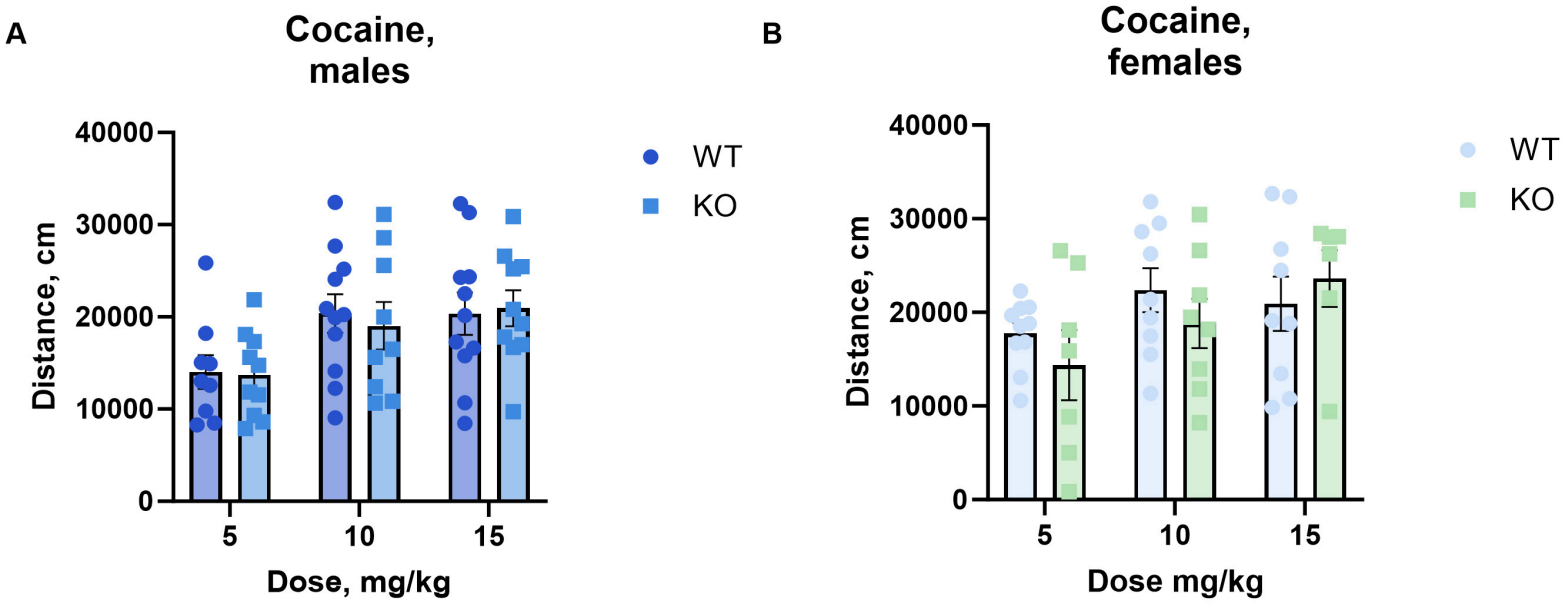
The absence of M_4_ mAChR in ChIs does not change male **(A)** or female **(B)** mice' locomotor response to cocaine at low to moderate doses. Both genotypes exhibit similar total distance moved (cm) in response to 5, 10, and 15 mg/kg cocaine doses, indicating comparable stimulant sensitivity in this range. Abscissa: Dose of cocaine, mg/kg. Ordinate: total distance (cm) measured in an open field for 1 h. *n* = 7–11 per group.

To determine the effect of cholinergic neuronal M_4_ mAChRs on cocaine-induced hyperactivity, we measured locomotor activity in an open field following the administration of increasing doses of cocaine. Male and female knockouts and their wild type littermates reacted similarly to cocaine doses from 5 to 15 mg/kg (Figure 2).

**Figure 3:**
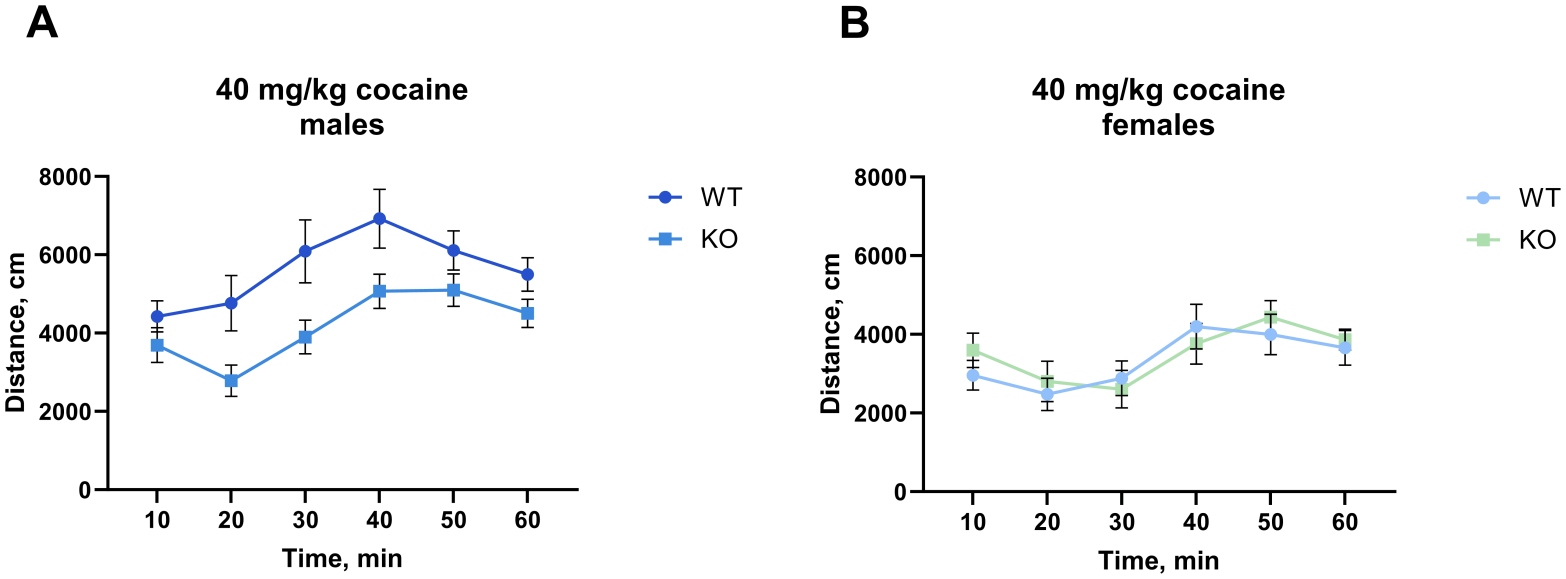
Knockout of M_4_ mAChR in ChIs attenuates response to a high cocaine dose (40 mg/kg) in male but not female mice. **(A)** Male KO mice display significantly reduced locomotor activity compared to WT males, suggesting a genotype-specific response at high doses of cocaine. *n* = 15–18 per group. **(B)** Female KO and WT mice show no difference in locomotor response. *n* = 8–10 per group. Distance in cm was analyzed for the 90 min after cocaine treatment. Abscissa: Distance (cm) moved. Ordinate: 10-min time bins.

Interestingly, at the highest cocaine dose tested (40 mg/kg), we found that the M_4_ receptor mutant male mice exhibited significantly less activity than their wild type littermates (*p* = 0.0084), however, we did not observe a similar decrease in females (Figure 3, for details of the statistical results see Supplementary Table 2a). The same cohort of male mice was scored for cocaine-induced stereotypy and the unpaired t-test showed no significant difference between genotypes (*p* = 0.37) (Supplementary Figure 3).

### 3.4 Scopolamine-induced locomotor activity

**Figure 4:**
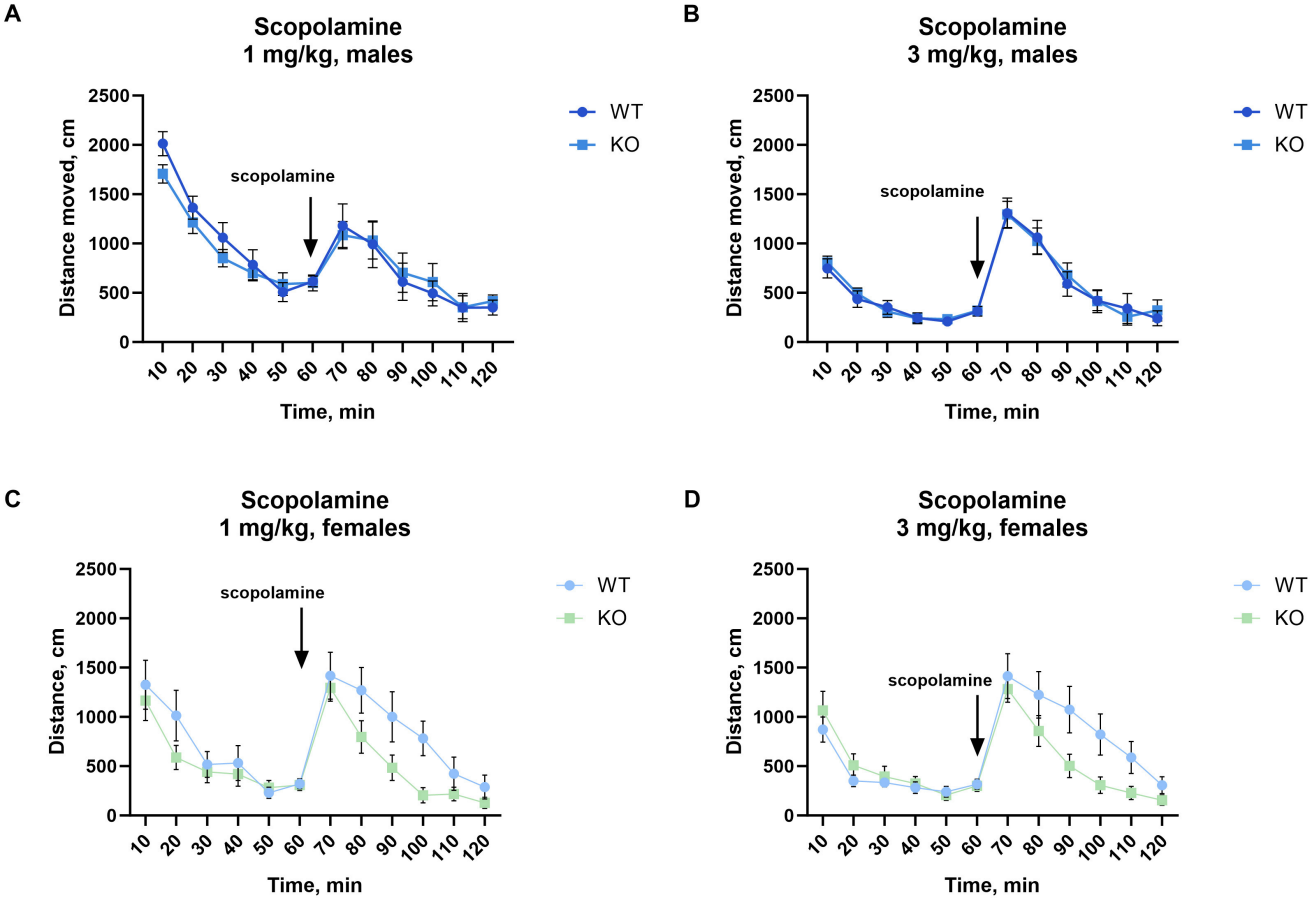
Female but not male mice lacking M_4_ mAChR in ChIs show an attenuated locomotor response to scopolamine. **(A, B)** Male KO and WT mice show no differences in scopolamine-induced activity at either 1 or 3 mg/kg, *n* = 11–13 per group. **(C)** Female KO mice show no significant difference from WT at 1 mg/kg, but **(D)** exhibit a significant reduction in locomotor response at 3 mg/kg. Results highlight a sex-specific effect of M_4_ mAChR deletion in cholinergic modulation by scopolamine. *n* = 9–14 per group. Distance in cm was analyzed for the 60 min after scopolamine treatment. Abscissa: Distance (cm) moved. Ordinate: 10-min time bins.

No significant difference in scopolamine-induced locomotion was found between WT and KO males, either after 1 mg/kg or after 3 mg/kg (Figures 4A, B). Most notably, female knockout mice showed significantly attenuated locomotion after 3 mg/kg (*p* = 0.04; Figure 4D) with a similar but not significant decrease at 1 mg/kg (*p* = 0.07; Figure 4C, for details of the statistical results see Supplementary Table 2).

### 3.5 Cocaine sensitization

**Figure 5:**
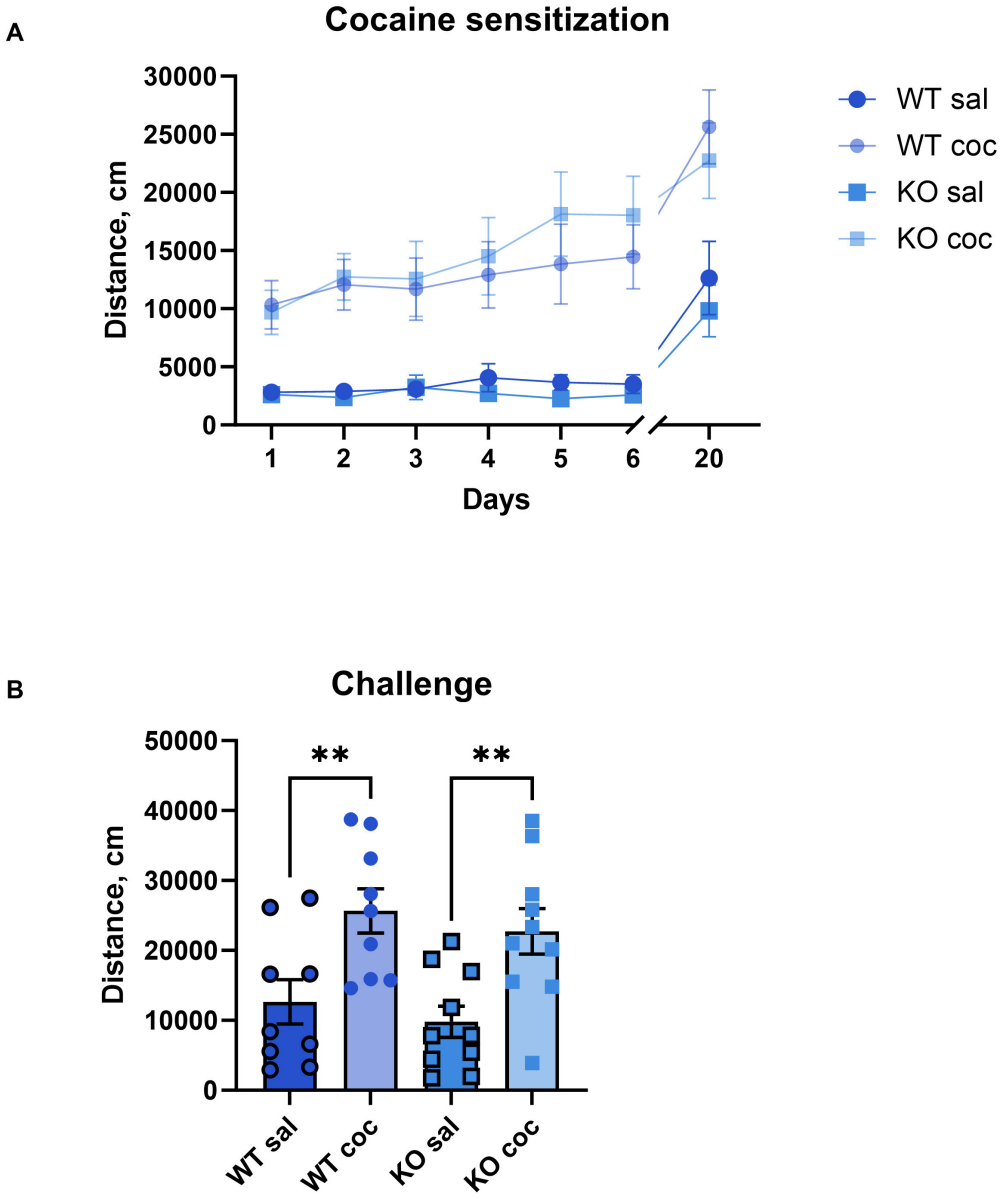
The development and expression of cocaine sensitization do not differ between KO mice and their wild type littermates. **(A)** Both genotypes show increased activity over days with repeated 15 mg/kg cocaine, indicating comparable sensitization development. Abscissa: Days of treatment. Ordinate: total distance (cm) measured in an open field for 1 h. **(B)** After a 2-week incubation, both groups display sensitized responses to a challenge dose, confirming no genotype effect on expression of cocaine sensitization. *n* = 9–10 per group. **Indicates *p* < 0.005. Abscissa: total distance (cm) measured in an activity chamber for 1 h. Ordinate: Groups.

In rodent models, repeated exposure to psychostimulants results in increased locomotor activity, known as behavioral sensitization, an effect that mirrors the prolonged drug sensitivity seen in human addiction and lasts several weeks or months post-administration (Robinson and Berridge, 1993). To study the role of M_4_ mAChRs on cholinergic neurons in the behavioral sensitization to cocaine, we administered daily doses of either saline or cocaine (15 mg/kg) to male wild type and knockout mice for 6 days. Similar to acute exposure, the repeated cocaine regimen significantly elevated locomotion in both groups compared to saline injections in both, WT and KO (Figure 5A). There was a significant effect of treatment (*p* = 0.0008) and day of treatment (*p* < 0.0001), indicating successful sensitization, but no significant effect of genotype and no significant interaction. On a challenge day (day 20), after an incubation period of 2 weeks, we administered a single dose of cocaine to all groups of mice, irrespective of whether they initially received saline or cocaine injections. This resulted in a significantly more pronounced locomotor response in the animals that had previously been injected with cocaine compared to the animals that had been injected with saline, regardless of genotype. Significant One-way ANOVA followed by Šídák's multiple comparisons test (WT sal vs. WT coc, *p* = 0.0096; KO sal vs. KO coc *p* = 0.0065) (Figure 5B, for details of the statistical results see Supplementary Table 2b). This indicates that knockout mice developed cocaine sensitization comparable to wild types.

### 3.6 Conditioned place preference

**Figure 6:**
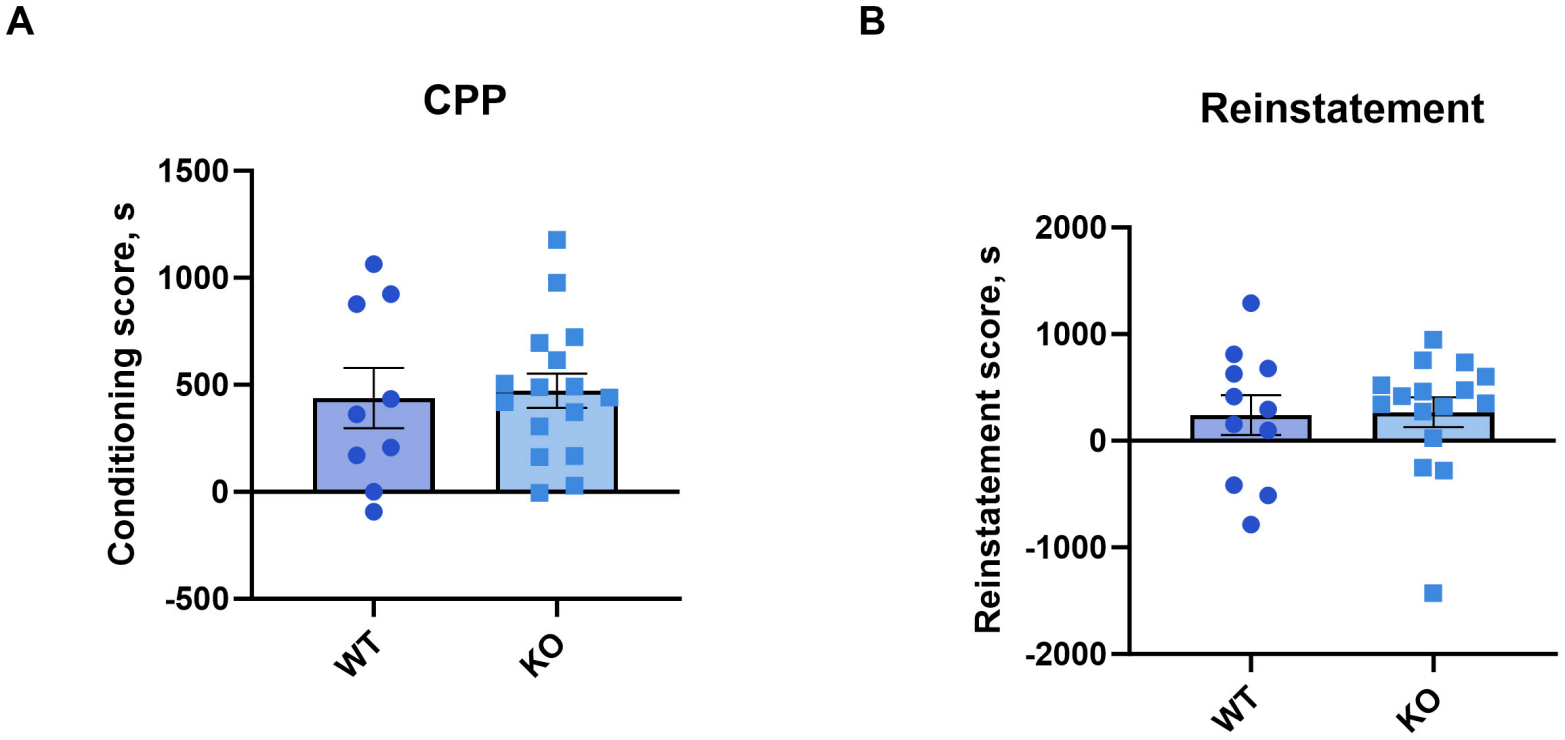
M_4_ mAChR deletion in ChIs does not alter the rewarding effects of cocaine, as assessed by conditioned place preference (CPP). **(A)** Conditioning scores and **(B)** reinstatement scores reveal similar cocaine-CPP in KO and WT male mice, indicating no genotype effect on cocaine reward. Data points represent individual animals (*n* = 11 WT, *n* = 16 KO).

A one-sample *t*-test revealed that both genotypes conditioning scores differed significantly from zero (WT: *p* = 0.0046; KO: *p* < 0.0001). The conditioning (unpaired *t*-test: *p* = 0.74) and reinstatement scores (*p* = 0.91) showed that cocaine produced a conditioned place preference in knockout male mice that was not different from their wild type littermates (Figures 6A, B). See Supplementary Figure 6 for time spent in the cocaine-paired compartment.

### 3.7 Food-reinforced operant behavior

**Figure 7:**
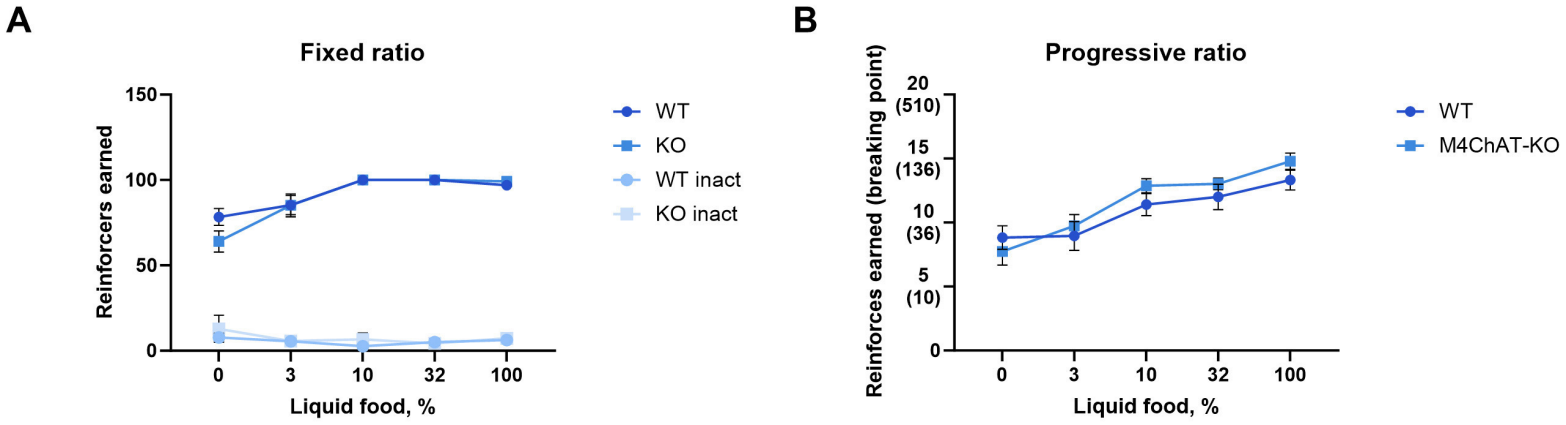
M_4_ mAChR deletion in ChIs does not affect operant learning in a food-reward paradigm. **(A)** Mutant male mice acquired normal levels of responding under a fixed ratio 1 schedule, suggesting preserved operant learning. Abscissa: Mean number of reinforcers earned during a 2-h session with a maximum of 100 per session. Ordinate: Concentration of liquid food diluted in water. Inact—inactive hole (number of inactive nose-poke responses per session). **(B)** Progressive ratio performance shows no significant differences, further supporting that M_4_ mAChR deletion does not affect reinforcement learning. Abscissa: Mean number of reinforcers earned during a maximum of 6-h session with breaking points in brackets. Ordinate: Concentration of liquid food diluted in water. Results suggest intact motivation and learning despite M_4_ receptor absence in ChIs.

In addition to classical conditioning, we assessed the contribution of M_4_ mAChRs on cholinergic neurons to operant tasks. We used the food self-administration paradigm under fixed and progressive ratio schedules. Again, wild type and knockout male mice successfully acquired the operant learning tasks, achieving comparable numbers of reinforcers per session, while nose pokes in the inactive hole diminished to minimal levels (Figures 7A, B for details of the statistical results see Supplementary Table 2c). The number of sessions needed to reach acquisition criteria in FR 1 and PR schedules did not differ between genotypes (*p* = 0.35 and *p* = 0.52, respectively; log-rank test). Moreover, no differences were found in the number of sessions required to extinguish in PR and FR schedules (*p* = 0.68 and *p* = 0.47, respectively; log-rank test) (see Table 2).

**Table 2:** Mean number of sessions required in an operant learning task to reach criteria in KO and WT mice.

**Criteria**	**KO**	**WT**
Liquid food FR 1 acquisition	2.5 ± 0.2 (15)	2.6 ± 0.3 (15)
Liquid food FR 1 extinction	2.2 ± 0.3 (15)	2.6 ± 0.3 (15)
Liquid food PR baseline	3.0 ± 0.3 (13)	3.4 ± 0.3 (14)
Liquid food PR extinction	3.0 ± 0.4 (13)	3.2 ± 0.4 (14)

Data are group means ± SEM, with the number of animals in brackets.

### 3.8 Haloperidol-induced catalepsy

Our study focused on the cataleptic effects induced by haloperidol in both control and knockout mice. We administered two doses of haloperidol (0.3 and 1 mg/kg, intraperitoneally) to the mice and assessed their cataleptic behavior at intervals of 30, 60, and 90 min (Supplementary Figure 4). Overall, ANOVA showed that both genotypes and sexes were similarly affected by haloperidol. No effect of genotype or genotype and time interaction was discovered for either dose or sex. Additionally, the *t*-test for scopolamine's ability to reverse catalepsy showed no significant differences between the genotypes.

## 4 Discussion

To explore the physiological significance of a specific subset of neuronal M_4_ mAChRs, mutant mice lacking M_4_ exclusively in cholinergic neurons were used. Subsequently, we subjected these knockout animals to a series of behavioral tests, with a primary focus on the motor, sensitizing, and rewarding effects of cocaine. Cholinergic neuron-specific depletion of the M_4_ receptor in knockout mice was confirmed using *in situ* hybridization. Results showed minimal Chrm4 and Chat mRNA co-expression in knockout mice, indicating effective depletion. First, basal phenotype assessment revealed no developmental deficits in knockout mice. Following administration of cocaine, both genotypes exhibited hyperlocomotion, with no differences observed at lower doses. However, at the highest cocaine dose tested, male knockout mice displayed significantly less activity compared to wild type littermates. Behavioral sensitization to cocaine was similar between knockout and wild type mice, with both groups showing increased locomotion upon repeated cocaine exposure. Additionally, conditioned place preference tests indicated no differences in the rewarding effects of cocaine between genotypes. In operant tasks assessing food-reinforced behavior knockout and wild type mice successfully acquired the tasks with comparable performance results. Furthermore, M_4_ receptor depletion did not affect haloperidol-induced catalepsy and scopolamine reversal of catalepsy but attenuated scopolamine-induced locomotion in a sex-dependent manner. Overall, these findings suggest that depletion of M_4_ receptor on cholinergic neurons does not significantly impact basal behavior or cocaine-induced hyperactivity but may modulate the response to high doses of cocaine in male mice and the response to scopolamine in female mice.

### 4.1 Genetic background considerations

On a general level, our findings contradict a previous characterization of an M_4−_ChAT-Cre knockout line (Klawonn et al., 2018). Despite normal acute response to cocaine and unchanged behavioral sensitization, the authors reported that M_4_-ChATCre mice showed significantly reduced CPP scores, indicating a failure to develop cocaine-induced place preference. A somewhat similar—albeit not significant—trend was observed with respect to palatable food CPP. Moreover, the authors showed that knockout mice lacked operant learning, demonstrated in a cocaine and palatable food operant runaway paradigm. They also observed unchanged basal locomotion, acute cocaine-induced hyperlocomotion, and cocaine sensitization, which is consistent with our findings. However, we were not able to replicate the reported deficits in cocaine CPP and attenuated food operant task performance in our knockout mice. One potential explanation for this inconsistency lies in the utilization of different driver lines to generate the two lines of specific knockouts. Two driver lines are available to drive Cre recombinase expression specifically in cholinergic neurons—ChAT(BAC)-Cre and ChAT(IRES)-Cre transgenic mouse lines. The ChAT(BAC)-Cre line utilizes bacterial artificial chromosome (BAC) technology (Ting and Feng, 2014), while the ChAT(IRES)-Cre line utilizes an internal ribosome entry site (IRES) to drive Cre recombinase expression under the control of the choline acetyltransferase (ChAT) promoter (Rossi et al., 2011). While we employed the ChAT (BAC)-Cre line, the previous study utilized the ChAT(IRES)-Cre line (Klawonn et al., 2018). Surprisingly, a comparative analysis of these two driver lines has suggested distinct phenotypes associated with the ChAT(IRES)-Cre-line, potentially influencing the observed behavioral responses (Chen et al., 2018). In particular, ChAT(IRES)-Cre transgenic (Tg/Tg) mice showed reduced generalized locomotor activity, attenuated operant food training, and resistance to nicotine-induced hypolocomotion together with decreased ChAT protein synthesis in the hippocampus. Another study revealed an alteration of ChAT, choline transporter (CHT), and vesicular acetylcholine transporter (VAChT) gene expression in the striatum of the transgenic ChAT(IRES)-Cre mice, which was accompanied by mild social disturbances and anxiety (Lhopitallier et al., 2022). It is possible that these alterations might have contributed to the discrepant results of our study and Klawonn et al. (2018), particularly to the observed differences in operant responding, as the runway task employed by Klawonn et al. is dependent on locomotor activity.

ChAT(BAC)-Cre transgenic mice showed no differences in basal locomotor activity, anxiety-related behavior, nicotine-induced hypolocomotion, drug-induced cataplexy, or operant food training (Chen et al., 2018). It is worth noting that the ChAT(BAC)-Cre driver line showed higher levels of ChAT and VAChT protein in the hippocampus despite normal behavior. Here, using Western blotting, we measured ChAT protein in the striatum of KO and WT mice, and no difference was found (see Supplementary Figure 5).

Given the phenotypic similarity between the driver line and knockout mice observed in the previous study (Klawonn et al., 2018), reflected in the attenuation of operant food training, we propose that the ChAT(IRES)-Cre driver line employed for manipulating M_4_ receptor expression played a substantial role in shaping the behavioral phenotype of the knockout mice, since our knockouts generated from the ChAT(BAC)-Cre line successfully acquired Pavlovian and operant conditioning. This is also consistent with a recent report demonstrating that the dynamics of dopamine in the striatum and its encoding of reward do not rely on acetylcholine release from cholinergic interneurons (Chantranupong et al., 2023).

However, environmental variables such as housing conditions, light cycles, and handling procedures could also contribute to variability in behavioral outcomes. Though we controlled for these factors within our protocol, subtle differences in stress levels or environmental enrichment may influence cholinergic and dopaminergic system responses, potentially affecting the replicability of findings across different laboratory settings. Therefore, a direct comparison of ChAT(BAC)-Cre and ChAT(IRES)-Cre driven M_4_ knockouts effect on Pavlovian and operant conditioning might be in order.

### 4.2 Relevance of findings

We cannot disregard the possibility of compensatory mechanisms occurring in the brains of knockout mice. Firstly, it is known that M_2_ receptors are co-expressed with M_4_ on cholinergic interneurons and can autoregulate acetylcholine dynamics (Zhang et al., 2002). A previous study has shown that the M_4_ receptor subtype is the main inhibitory muscarinic autoreceptor (Zhang et al., 2002). Additionally, immunofluorescence microscopy showed that M_2_ receptors localized on cholinergic terminals in the striatum do not redistribute in response to the absence of M_4_ receptors (Zhang et al., 2002). Therefore, it is not likely that our results are due to a compensatory change in M_2_ receptor expression. However, another study involving mAChR subtype-null mice demonstrated that in the ventral striatum, only M_4_ autoreceptors are necessary for the indirect control of dopamine release, whereas, in the dorsal striatum, both M_2_ and M_4_ are required (Threlfell et al., 2010). Overall, it is suggested that both M_4_ and M_2_ receptors can contribute to the autoregulation of acetylcholine dynamics and their relative importance varies by brain region.

Converging evidence indicates that multiple feedback mechanisms can regulate acetylcholine tone in the striatum (Lim et al., 2014). This implies that the influence of M_4_ receptors on cholinergic interneurons may be relatively insignificant under normal conditions but may become apparent when the system is subjected to intense external stimuli, such as high doses of cocaine. For example, dopamine acting at D_2_ inhibitory receptors expressed on ChIs, decreases pacemaking activity and reduces the release of acetylcholine in the striatum, similar to the effects of M_4_ signaling (Kharkwal et al., 2016; Maurice et al., 2004). Lewis et al. demonstrated that the absence of D_2_R signaling in cholinergic neurons reduces the response to cocaine in a dose-dependent manner (Lewis et al., 2020), which is similar to what we observed in our KO mice, albeit at higher levels of stimulation. Specifically, we found a diminished locomotor response to 40 mg/kg cocaine in knockout male mice. To examine whether this is due to the differential induction of stereotypic behavior at this high dose of cocaine (Tolliver and Carney, 1994), we analyzed the videos for cocaine-induced stereotypy to address this possibility and found that decreased locomotion was not driven by repetitive behavior such as sniffing, grooming, circling, or rearing (see Supplementary Figure 4). We speculate that the observed decreased cocaine-induced hyperactivity may be attributed to elevated acetylcholine levels resulting from the absence of M_4_-inhibitory feedback. This elevation could potentially activate M_4_ receptors on dSPNs and corticostriatal and thalamic fibers, thereby inhibiting the direct striatonigral pathway and consequently preventing a full motor response to psychostimulants. However, further investigation is needed to elucidate the precise synaptic and cellular mechanisms underlying the diminished response to a high dose of psychostimulant in M_4_ autoreceptor knockout male mice.

Additionally, data reveal an alternative source of acetylcholine in the striatum originating from the pedunculopontine nucleus (PPN) and laterodorsal tegmental nucleus (LDT) (Dautan et al., 2014). Brainstem cholinergic terminals that arise from the PPN and LDT also express M_4_ as autoreceptors, and it is known that these terminals project to CPu and NAc, where they modulate the activity of striatal ChIs (Dautan et al., 2020). In the present study, we have focused on striatal cholinergic cells as the main modulators of striatum output (Brimblecombe et al., 2018). However, cholinergic neurons in the PPN and LDT may also play a role in the behavioral outcome of the present study. Moreover, the basal forebrain contains a significant proportion of cholinergic neurons which are the major source of acetylcholine in the cortex, hippocampus and amygdala (Woolf, 1991). These cholinergic neurons are crucial regulators of cognitive functions and were shown to be critical in encoding sensory cues and behavioral reinforcement (Blake and Boccia, 2018; Robert et al., 2021). These cholinergic neurons also express M_2_ and M_4_ presynaptically but the M_2_ subtype seems to be the predominant autoreceptor in the basal forebrain nuclei (Levey et al., 1991; Zhang et al., 2002). Despite the limited evidence regarding the role of presynaptic M_4_ receptors in basal forebrain cholinergic neurons we also must consider the possible contribution of this subpopulation of M_4_ autoreceptors to the observed phenotype.

In the present study, we assessed behaviors that recruit both the ventral and dorsal striatum. Given the differential significance of presynaptic M_4_ receptors in those areas (Threlfell et al., 2010), we speculate that the lack of differences observed in the operant task in M_4_ knockout mice could be due to the task's reliance on the proper functioning of the dorsomedial striatum where M_2_ receptors seem to be more important for the modulation of dopaminergic signaling. Cocaine-induced hyperlocomotion, behavioral sensitization, and CPP are primarily dependent on nucleus accumbens functioning, where M_4_ autoreceptors appear to be more critical (Threlfell et al., 2010). Indeed, we observed a difference in the effect of high doses of cocaine or scopolamine on hyperlocomotion, however the rewarding and sensitizing effects of cocaine were not affected.

Previous studies have reported a dose-dependent increase in locomotor activity induced by the muscarinic antagonist scopolamine, which was notably absent in global M_4_ knockout mice (Moehle et al., 2021), suggesting the involvement of these receptors in the behavioral response to scopolamine. THP, another non-selective mAChR antagonist commonly used in dystonia treatment (Thenganatt and Jankovic, 2014), has been shown to partially exert its effects through M_4_ autoreceptors, enhancing dopamine release (Downs et al., 2019). Furthermore, Downs and colleagues reported that a selective M_4_ subtype mAChR antagonist (VU6021625) enhances DA release by specifically blocking M_4_ receptors on cholinergic interneurons (Downs et al., 2022). Our assessment of locomotor activity in an open field following scopolamine treatment yielded similar results, with female knockout mice exhibiting diminished scopolamine-induced hyperlocomotion at 3 mg/kg dose with a similar but not significant trend at 1 mg/kg. However, this was not observed in the male mice.

Until recently, the predominant focus in rodent studies has been on males. In our investigation, both male and female subjects were included. Our findings reveal that only male knockout subjects displayed reduced cocaine-induced hyperlocomotion, whereas females did not differ from wild type littermates. This might be explained by sex differences in the dopaminergic system (Woodcock et al., 2020) however, the precise mechanism by which deletion of the M_4_ muscarinic autoreceptor might have sexually dimorphic effects on cocaine-induced hyperlocomotion needs further examination. Conversely, only female mice showed changes in scopolamine-induced locomotion, while male mice displayed no differences between genotypes. Unfortunately, in the present study, only male M_4_ autoreceptor deficient mice were tested in the operant conditioning task. Given the specific sensitivity of female KO mice to cholinergic challenge and the known involvement of the cholinergic system in learning and memory it would have been relevant to also assess female mice in this task.

Previous research has shown that sex hormones influence M_4_ expression. Ovariectomy, for example, led to the upregulation of M_4_ muscarinic receptors across various brain regions, including the hippocampus, hypothalamus, and frontal cortex (El-Bakri et al., 2002). Estrogen replacement therapy restored M_4_ levels to baseline (El-Bakri et al., 2002). Studies have indicated that sex hormones can impact circadian rhythms (Bailey and Silver, 2014) and it has also been reported that global M_4_ deletion increases motor activity exclusively in females during the dark phase, highlighting sex-related differences that were not observed in males (Valuskova et al., 2018). Overall, while the precise nature of these sex differences remains unclear, earlier reports indicate that sex hormones and circadian rhythms could mediate some of the effects observed in our study (El-Bakri et al., 2002; Valuskova et al., 2018).

### 4.3 Future directions

As mentioned above, logical follow-up to this study would involve including female mice in the operant conditioning tasks to assess potential sex-specific effects of M_4_ receptor deletion on reinforcement learning and operant responding. Given the observed differences in scopolamine-induced locomotion between male and female knockout mice, testing females in operant paradigms could reveal further sex-dependent effects on motivation and learning, particularly given the role of acetylcholine in these cognitive processes. In addition, expanding on the pharmacological scope of this study by testing other agents that engage the dopaminergic and cholinergic systems differently and more specifically than cocaine and scopolamine might be of relevance. For example, examining the effects of drugs selective for specific dopamine or muscarinic receptor subtypes could provide insight into the specific roles of M_4_ autoreceptors in modulating dopamine-dependent and cholinergic behaviors across a broader range of stimuli.

## 5 Conclusion

Our findings are in discrepancy with previous research data that demonstrated impaired learning in knockout mice lacking M_4_ receptors on cholinergic neurons (Klawonn et al., 2018). Contrary to the earlier study, we observed no significant alterations in the rewarding properties of cocaine as well as in operant learning in knockout mice. However, we have seen sex-specific effects of M_4_ autoreceptor deletion on cocaine and scopolamine induced locomotion. Overall, these findings highlight the nuanced, sex-specific roles of M_4_ autoreceptors in modulating behavioral responses to pharmacological challenges, suggesting that targeted modulation of M_4_ receptors could serve as a promising strategy for developing drugs that address sex-specific behavioral disorders. By tailoring therapeutic approaches to the distinct neurochemical responses observed in male and female subjects, such interventions could enhance efficacy and reduce side effects in treating conditions like schizophrenia, substance use disorders, Parkinson's disease, and other neuropsychiatric disorders with sex-dependent characteristics.

## Data Availability

The raw data supporting the conclusions of this article will be made available by the authors, without undue reservation.

## References

[B1] Bailey M. Silver R. (2014). Sex differences in circadian timing systems: implications for disease. Front. Neuroendocrinol. 35, 111–139. 10.1016/j.yfrne.2013.11.003 24287074 PMC4041593

[B2] Bankhead P. Loughrey M. B. Fernández J. A. Dombrowski Y. McArt D. G. Dunne P. D.. (2017). QuPath: Open source software for digital pathology image analysis. Scient. Reports 7, 1–7. 10.1038/s41598-017-17204-5 29203879 PMC5715110

[B3] Becker J. B. (1999). Gender differences in dopaminergic function in striatum and nucleus accumbens. Pharmacol. Biochem. Behav. 64, 803–812. 10.1016/S0091-3057(99)00168-9 10593204

[B4] Bennett B. D. Callaway J. C. Wilson C. J. (2000). Intrinsic membrane properties underlying spontaneous tonic firing in neostriatal cholinergic interneurons. J. Neurosci. 20, 8493–8503. 10.1523/JNEUROSCI.20-22-08493.2000 11069957 PMC6773196

[B5] Bernard V. Levey A. I. Bloch B. (1999). Regulation of the subcellular distribution of m4 muscarinic acetylcholine receptors in striatal neurons *in vivo* by the cholinergic environment: evidence for regulation of cell surface receptors by endogenous and exogenous stimulation. J. Neurosci. 19:10237. 10.1523/JNEUROSCI.19-23-10237.1999 10575021 PMC6782421

[B6] Blake M. G. Boccia M. M. (2018). Basal forebrain cholinergic system and memory. Curr. Top. Behav. Neurosci. 37, 253–273. 10.1007/7854_2016_467 28213811

[B7] Brady A. E. Jones C. K. Bridges T. M. Kennedy J. P. Thompson A. D. Heiman J. U.. (2008). Centrally active allosteric potentiators of the M4 muscarinic acetylcholine receptor reverse amphetamine-induced hyperlocomotor activity in rats. J. Pharmacol. Exp. Ther. 327:941. 10.1124/jpet.108.140350 18772318 PMC2745822

[B8] Brimblecombe K. R. Threlfell S. Dautan D. Kosillo P. Mena-Segovia J. Cragg S. J. (2018). Targeted activation of cholinergic interneurons accounts for the modulation of dopamine by striatal nicotinic receptors. ENeuro 5, 397–414. 10.1523/ENEURO.0397-17.2018 30406189 PMC6220583

[B9] Byun N. E. Grannan M. Bubser M. Barry R. L. Thompson A. Rosanelli J.. (2014). Antipsychotic drug-like effects of the selective m4 muscarinic acetylcholine receptor positive allosteric modulator VU0152100. Neuropsychopharmacology 39:1578. 10.1038/npp.2014.2 24442096 PMC4023154

[B10] Calabresi P. Centonze D. Pisani A. Sancesario G. North R. A. Bernardi G. (1998). Muscarinic IPSPs in rat striatal cholinergic interneurones. J. Physiol. 510, 421–427. 10.1111/j.1469-7793.1998.421bk.x 9705993 PMC2231046

[B11] Caulfield M. P. (1993). Muscarinic receptors–characterization, coupling and function. Pharmacol. Therapeut. 58, 319–379. 10.1016/0163-7258(93)90027-B 7504306

[B12] Chantranupong L. Beron C. C. Zimmer J. A. Wen M. J. Wang W. Sabatini B. L. (2023). Dopamine and glutamate regulate striatal acetylcholine in decision-making. Nature 621, 577–585. 10.1038/s41586-023-06492-9 37557915 PMC10511323

[B13] Chen E. Lallai V. Sherafat Y. Grimes N. P. Pushkin A. N. Fowler J. P.. (2018). Altered baseline and nicotine-mediated behavioral and cholinergic profiles in ChAT-Cre mouse lines. J. Neurosci. 38:2177. 10.1523/JNEUROSCI.1433-17.2018 29371319 PMC5830509

[B14] Chuhma N. Mingote S. Moore H. Rayport S. (2014). Dopamine neurons control striatal cholinergic neurons via regionally heterogeneous dopamine and glutamate signaling. Neuron 81:901. 10.1016/j.neuron.2013.12.027 24559678 PMC3933825

[B15] Cui G. Jun S. B. Jin X. Pham M. D. Vogel S. S. Lovinger D. M.. (2013). Concurrent activation of striatal direct and indirect pathways during action initiation. Nature 494, 238–242. 10.1038/nature11846 23354054 PMC4039389

[B16] Dall C. Weikop P. Dencker D. Molander A. C. Wörtwein G. Conn P. J.. (2017). Muscarinic receptor M4 positive allosteric modulators attenuate central effects of cocaine. Drug Alcohol Depend. 176, 154–161. 10.1016/j.drugalcdep.2017.03.014 28544993 PMC6423356

[B17] Dautan D. Huerta-Ocampo I. Gut N. K. Valencia M. Kondabolu K. Kim Y.. (2020). Cholinergic midbrain afferents modulate striatal circuits and shape encoding of action strategies. Nat. Commun. 11:1. 10.1038/s41467-020-15514-3 32269213 PMC7142106

[B18] Dautan D. Huerta-Ocampo I. Witten I. B. Deisseroth K. Paul Bolam J. Gerdjikov T.. (2014). A major external source of cholinergic innervation of the striatum and nucleus accumbens originates in the brainstem. J. Neurosci. 34, 4509–4518. 10.1523/JNEUROSCI.5071-13.2014 24671996 PMC3965779

[B19] De La Cour C. Sørensen G. Wortwein G. Weikop P. Dencker D. Fink-Jensen A.. (2015). Enhanced self-administration of alcohol in muscarinic acetylcholine M4 receptor knockout mice. Eur. J. Pharmacol. 746, 1–5. 10.1016/j.ejphar.2014.10.050 25445043

[B20] Dencker D. Weikop P. Sørensen G. Woldbye D. P. D. Wörtwein G. Wess J.. (2012). An allosteric enhancer of M4 muscarinic acetylcholine receptor function inhibits behavioral and neurochemical effects of cocaine. Psychopharmacology 224:2. 10.1007/s00213-012-2751-8 22648127 PMC3914671

[B21] Ding J. Guzman J. N. Tkatch T. Chen S. Goldberg J. A. Ebert P. J.. (2006). RGS4-dependent attenuation of M4 autoreceptor function in striatal cholinergic interneurons following dopamine depletion. Nat. Neurosci. 9, 832–842. 10.1038/nn1700 16699510

[B22] Downs A. M. Donsante Y. Jinnah H. A. Hess E. J. (2022). Blockade of M4 muscarinic receptors on striatal cholinergic interneurons normalizes striatal dopamine release in a mouse model of TOR1A dystonia. Neurobiol. Dis. 168:105699. 10.1016/j.nbd.2022.105699 35314320

[B23] Downs A. M. Fan X. Donsante C. Jinnah H. A. Hess E. J. (2019). Trihexyphenidyl rescues the deficit in dopamine neurotransmission in a mouse model of DYT1 dystonia. Neurobiol. Dis. 125, 115–122. 10.1016/j.nbd.2019.01.012 30707939 PMC6863078

[B24] El-Bakri N. K. Adem A. Suliman I. A. Mulugeta E. Karlsson E. Lindgren J. U.. (2002). Estrogen and progesterone treatment: Effects on muscarinic M4 receptor subtype in the rat brain. Brain Res. 948, 131–137. 10.1016/S0006-8993(02)02962-1 12383964

[B25] Fink-Jensen A. Schmidt L. S. Dencker D. Schülein C. Wess J. Wörtwein G.. (2011). Antipsychotic-induced catalepsy is attenuated in mice lacking the M4 muscarinic acetylcholine receptor. Eur. J. Pharmacol. 656:39. 10.1016/j.ejphar.2011.01.018 21269601 PMC3896864

[B26] Foster D. J. Wilson J. M. Remke D. H. Mahmood M. S. Uddin M. J. Wess J.. (2016). Antipsychotic-like Effects of M4 Positive Allosteric Modulators Are Mediated by CB2 Receptor-dependent inhibition of dopamine release. Neuron 91, 1244–1252. 10.1016/j.neuron.2016.08.017 27618677 PMC5033724

[B27] Gomeza J. Zhang L. Kostenis E. Felder C. Bymaster F. Brodkin J.. (1999). Enhancement of D1 dopamine receptor-mediated locomotor stimulation in M4 muscarinic acetylcholine receptor knockout mice. Proc. Natl. Acad. Sci. USA. 96:10483. 10.1073/pnas.96.18.10483 10468635 PMC17915

[B28] Gong S. Doughty M. Harbaugh C. R. Cummins A. Hatten M. E. Heintz N.. (2007). Targeting Cre recombinase to specific neuron populations with bacterial artificial chromosome constructs. J. Neurosci. 27:9817. 10.1523/JNEUROSCI.2707-07.2007 17855595 PMC6672645

[B29] Itzhak Y. Martin J. L. (2000). Scopolamine inhibits cocaine-conditioned but not unconditioned stimulant effects in mice. Psychopharmacology 152, 216–223. 10.1007/s002130000537 11057526

[B30] Jeon J. Dencker D. Wörtwein G. Woldbye D. P. D. Cui Y. Davis A. A.. (2010). A subpopulation of neuronal M4 muscarinic acetylcholine receptors plays a critical role in modulating dopamine-dependent behaviors. J. Neurosci. 30, 2396–2405. 10.1523/JNEUROSCI.3843-09.2010 20147565 PMC2824442

[B31] Kawaguchi Y. Wilson C. J. Augood S. J. Emson P. C. (1995). Striatal interneurones: chemical, physiological and morphological characterization. Trends Neurosci. 18, 527–535. 10.1016/0166-2236(95)98374-8 8638293

[B32] Kharkwal G. Brami-Cherrier K. Lizardi-Ortiz J. E. Nelson A. B. Ramos M. Del Barrio D.. (2016). Parkinsonism driven by antipsychotics originates from dopaminergic control of striatal cholinergic interneurons. Neuron 91, 67–78. 10.1016/j.neuron.2016.06.014 27387649 PMC4939839

[B33] Klawonn A. M. Wilhelms D. B. Lindström S. H. Singh A. K. Jaarola M. Wess J.. (2018). Muscarinic M4 receptors on cholinergic and dopamine D1 receptor-expressing neurons have opposing functionality for positive reinforcement and influence impulsivity. Front. Mol. Neurosci. 11:139. 10.3389/fnmol.2018.00139 29740282 PMC5928231

[B34] Kravitz A. V. Freeze B. S. Parker P. R. L. Kay K. Thwin M. T. Deisseroth K.. (2010). Regulation of Parkinsonian motor behaviours by optogenetic control of basal ganglia circuitry. Nature 466, 622–626. 10.1038/nature09159 20613723 PMC3552484

[B35] Kravitz A. V. Tye L. D. Kreitzer A. C. (2012). Distinct roles for direct and indirect pathway striatal neurons in reinforcement. Nat. Neurosci. 15, 816–818. 10.1038/nn.3100 22544310 PMC3410042

[B36] Kreitzer A. C. Malenka R. C. (2008). Striatal plasticity and basal ganglia circuit function. Neuron 60:543. 10.1016/j.neuron.2008.11.005 19038213 PMC2724179

[B37] Lalonde R. Filali M. Strazielle C. (2021). SHIRPA as a neurological screening battery in mice. Current Protocols 1:5. 10.1002/cpz1.135 34000103

[B38] Levey A. I. Kitt C. A. Simonds W. F. Price D. L. Brann M. R. (1991). Identification and localization of muscarinic acetylcholine receptor proteins in brain with subtype-specific antibodies. J. Neurosci. 11, 3218–3226. 10.1523/JNEUROSCI.11-10-03218.1991 1941081 PMC6575445

[B39] Lewis R. G. Serra M. Radl D. Gori M. Tran C. Michalak S. E.. (2020). Dopaminergic control of striatal cholinergic interneurons underlies cocaine-induced psychostimulation. Cell Rep. 31:107527. 10.1016/j.celrep.2020.107527 32320647

[B40] Lhopitallier C. Perrault C. Chauveau F. Saurini F. Berrard S. Granon S.. (2022). Characterization of social behavior in young and middle-aged ChAT-IRES-Cre mouse. PLoS ONE 17:e272141. 10.1371/journal.pone.0272141 35925937 PMC9352053

[B41] Lim S. A. O. Kang U. J. McGehee D. S. (2014). Striatal cholinergic interneuron regulation and circuit effects. Front. Synaptic Neurosci. 6:22. 10.3389/fnsyn.2014.00022 25374536 PMC4204445

[B42] Maurice N. Mercer J. Chan C. S. Hernandez-Lopez S. Held J. Tkatch T.. (2004). D2 dopamine receptor-mediated modulation of voltage-dependent Na+ channels reduces autonomous activity in striatal cholinergic interneurons. J. Neurosci. 24, 10289–10301. 10.1523/JNEUROSCI.2155-04.2004 15548642 PMC6730305

[B43] Moehle M. S. Bender A. M. Dickerson J. W. Foster D. J. Qi A. Cho H. P.. (2021). Discovery of the first selective M4Muscarinic acetylcholine receptor antagonists with *in vivo* antiparkinsonian and antidystonic efficacy. ACS Pharmacol. Transl. Sci. 4, 1306–1321. 10.1021/acsptsci.0c00162 34423268 PMC8369681

[B44] Moran S. P. Maksymetz J. Conn P. J. (2019). Targeting muscarinic acetylcholine receptors for the treatment of psychiatric and neurological disorders. Trends Pharmacol. Sci. 40, 1006–1020. 10.1016/j.tips.2019.10.007 31711626 PMC6941416

[B45] Myslivecek J. (2021). Two players in the field: hierarchical model of interaction between the dopamine and acetylcholine signaling systems in the striatum. Biomedicines 9, 1–19. 10.3390/biomedicines9010025 33401461 PMC7824505

[B46] Nair A. G. Castro L. R. V. El Khoury M. Gorgievski V. Giros B. Tzavara E. T.. (2019). The high efficacy of muscarinic M4 receptor in D1 medium spiny neurons reverses striatal hyperdopaminergia. Neuropharmacology 146, 74–83. 10.1016/j.neuropharm.2018.11.029 30468798

[B47] Pancani T. Bolarinwa C. Smith Y. Lindsley C. W. Conn P. J. Xiang Z. (2014). M4 mAChR-mediated modulation of glutamatergic transmission at corticostriatal synapses. ACS Chem Neurosci. 5, 318–324. 10.1021/cn500003z 24528004 PMC3990947

[B48] Paul S. M. Yohn S. E. Popiolek M. Miller A. C. Felder C. C. (2022). Muscarinic acetylcholine receptor agonists as novel treatments for schizophrenia. Am. J. Psychiatry 179, 611–627. 10.1176/appi.ajp.21101083 35758639

[B49] Poppi L. A. Ho-Nguyen K. T. Shi A. Daut C. T. Tischfield M. A. (2021). Recurrent implication of striatal cholinergic interneurons in a range of neurodevelopmental, neurodegenerative, and neuropsychiatric disorders. Cells 10:907. 10.3390/cells10040907 33920757 PMC8071147

[B50] Robert B. Kimchi E. Y. Watanabe Y. Chakoma T. Jing M. Li Y.. (2021). A functional topography within the cholinergic basal forebrain for encoding sensory cues and behavioral reinforcement outcomes. Elife 10:e69514. 10.7554/eLife.69514.sa2 34821218 PMC8654357

[B51] Robinson T. E. Berridge K. C. (1993). The neural basis of drug craving: an incentive-sensitization theory of addiction. Brain Res. Rev. 18, 247–291. 10.1016/0165-0173(93)90013-P 8401595

[B52] Rossi J. Balthasar N. Olson D. Scott M. Berglund E. Lee C. E.. (2011). Melanocortin-4 receptors expressed by cholinergic neurons regulate energy balance and glucose homeostasis. Cell Metab. 13, 195–204. 10.1016/j.cmet.2011.01.010 21284986 PMC3033043

[B53] Sanberg P. R. Bunsey M. D. Giordano M. Norman A. B. (1988). The catalepsy test: its ups and downs. Behav. Neurosci. 102, 748–759. 10.1037/0735-7044.102.5.748 2904271

[B54] Schmidt L. S. Thomsen M. Weikop P. Dencker D. Wess J. Woldbye D. P. D.. (2011). Increased cocaine self-administration in m4 muscarinic acetylcholine receptor knockout mice. Psychopharmacology 216, 367–378. 10.1007/s00213-011-2225-4 21373792 PMC3899540

[B55] Tanimura A. Pancani T. Lim S. A. O. Tubert C. Melendez A. E. Shen W.. (2018). Striatal cholinergic interneurons and Parkinson's disease. Eur. J. Neurosci. 47:1148. 10.1111/ejn.13638 28677242 PMC6074051

[B56] Thenganatt M. A. Jankovic J. (2014). Treatment of dystonia. Neurotherapeutics 11, 139–152. 10.1007/s13311-013-0231-4 24142590 PMC3899473

[B57] Thomsen M. (2014). Locomotor activating effects of cocaine and scopolamine combinations in rats: isobolographic analysis. Behav. Pharmacol. 25:259. 10.1097/FBP.0000000000000043 24769455 PMC4090251

[B58] Thomsen M. Han D. D. Gu H. H. Caine S. B. (2009). Lack of cocaine self-administration in mice expressing a cocaine-insensitive dopamine transporter. J. Pharmacol. Exp. Ther. 331, 204–211. 10.1124/jpet.109.156265 19602552 PMC2766230

[B59] Threlfell S. Clements M. A. Khodai T. Pienaar I. S. Exley R. Wess J.. (2010). Striatal muscarinic receptors promote activity dependence of dopamine transmission via distinct receptor subtypes on cholinergic interneurons in ventral versus dorsal striatum. J. Neuroscience 30:3398. 10.1523/JNEUROSCI.5620-09.2010 20203199 PMC2866006

[B60] Ting J. T. Feng G. (2014). Recombineering strategies for developing next generation BAC transgenic tools for optogenetics and beyond. Front. Behav. Neurosci. 8:111. 10.3389/fnbeh.2014.00111 24772073 PMC3982106

[B61] Tolliver B. K. Carney J. M. (1994). Sensitization to stereotypy in DBA/2J but not C57BL/6J mice with repeated cocaine. Pharmacol. Biochem. Behav. 48, 169–173. 10.1016/0091-3057(94)90513-4 8029287

[B62] Valuskova P. Forczek S. T. Farar V. Myslivecek J. (2018). The deletion of M4 muscarinic receptors increases motor activity in females in the dark phase. Brain Behav. 8:8. 10.1002/brb3.1057 29978954 PMC6085911

[B63] Walker L. C. Lawrence A. J. (2020). Allosteric modulation of muscarinic receptors in alcohol and substance use disorders. Adv. Pharmacol. 88, 233–275. 10.1016/bs.apha.2020.01.003 32416869

[B64] Wieland S. Du D. Oswald M. J. Parlato R. Köhr G. Kelsch W. (2014). Phasic dopaminergic activity exerts fast control of cholinergic interneuron firing via sequential NMDA, D2, and D1 receptor activation. J. Neurosci. 34, 11549–11559. 10.1523/JNEUROSCI.1175-14.2014 25164653 PMC6608407

[B65] Woodcock E. A. Zakiniaeiz Y. Morris E. D. Cosgrove K. P. (2020). Sex and the dopaminergic system: Insights from addiction studies. Handb. Clin. Neurol. 175, 141–165. 10.1016/B978-0-444-64123-6.00011-4 33008522 PMC11267480

[B66] Woolf N. J. (1991). Cholinergic systems in mammalian brain and spinal cord. Prog. Neurobiol. 37, 475–524. 10.1016/0301-0082(91)90006-M 1763188

[B67] Yan Z. Surmeier D. J. (1996). Muscarinic (m2/m4) receptors reduce N- and P-type Ca2+ currents in rat neostriatal cholinergic interneurons through a fast, membrane-delimited, G-protein pathway. J. Neurosci. 16, 2592–2604. 10.1523/JNEUROSCI.16-08-02592.1996 8786435 PMC6578763

[B68] Zhang W. Basile A. S. Gomeza J. Volpicelli L. A. Levey A. I. Wess J. (2002). Characterization of central inhibitory muscarinic autoreceptors by the use of muscarinic acetylcholine receptor knock-out mice. J. Neurosci. 22, 1709–1717. 10.1523/JNEUROSCI.22-05-01709.2002 11880500 PMC6758851

