# Supplementary material for: A sex-specific effect of M4 muscarinic cholinergic autoreceptor deletion on locomotor stimulation by cocaine and scopolamine
Source: Front Mol Neurosci. 2024 Dec 16;17:1451010. doi: 10.3389/fnmol.2024.1451010 (PMC11683150; doi:10.3389/fnmol.2024.1451010)
Supplement: Supplementary file 1 [file Data_Sheet_1.docx]

Supplementary Material

## Stereotaxic surgery

Surgical procedures were conducted under isoflurane anesthesia on both wild type (WT) and knockout (KO) mice (n=2 for each genotype). Before surgery, the mice were given 10 mg/kg carprofen sc. along with lidocaine applied topically at the incision site. Mice received a unilateral injection in the dorsal striatum with 500 nl of Cre-dependent AAV9. The plasmid used, pAAV-Ef1a-DIO hChR2(E123T/T159C)-EYFP, was kindly provided by Karl Deisseroth (Addgene plasmid # 35509-AAV9; http://n2t.net/addgene:35509; RRID: Addgene_35509). The mice were administered carprofen and antibiotics for two days post-surgically. Two weeks after surgery, animals were transcardially perfused with PBS followed by 4% PFA in PBS, brains were removed and immersed in 4% PFA overnight, followed by 30% sucrose in PBS for 24 hours. Later, the striatum region was cut into 40 μm sections on a cryostat, which were then mounted and coverslipped. Images were captured with Zeiss Axiovert Z1.

## Scoring of stereotypic behavior in male knockout and wild type mice after 40 mg/kg cocaine in an open field

Male wild type and knockout mice were initially habituated to the open field arena for 30 minutes before receiving an injection of 40 mg/kg cocaine with subsequent 60-minute recording. Stereotypies were scored as previously described (Creese & Iversen, 1973). Stereotypies were assessed for 1-min periods, every 5 min, during the first 30 minutes, and then every 10 min. The scoring criteria for stereotypies were as follows: 0: asleep or stationary 1: active 2: predominantly active with intermittent bursts of stereotyped sniffing or rearing (e.g., grooming, head-bobbing) 3: stereotyped activity, such as circling or following a fixed path in the cage 4: stereotyped sniffing or rearing behavior maintained in one location 5: stereotyped behavior in one location accompanied by bursts of gnawing, licking, or nail-biting 6: continuous gnawing, licking, or nail-biting.

## Western blotting

The striatal brain tissue from knockout and wild type mice (n=6 per group) was homogenized in NPER lysis buffer (catalog #87792, Thermo Fisher Scientific, Waltham, Massachusetts, USA) mixed with protease (catalog #P8340, Sigma-Aldrich) and phosphatase inhibitors (catalog #78420, Thermo Fisher Scientific). Protein concentration for each sample was determined using a Bradford Assay (catalog #B6916, Sigma-Aldrich). 10 µg of proteins were mixed with 4x loading buffer (catalog #928-40004, LI-COR Biosciences, Bad Homburg, Germany) and DTT, separated by 4-12% Bis-Tris Protein Gels (catalog #NP0329BOX, Thermo Fisher Scientific), and transferred to a nitrocellulose membrane (catalog #926-31092, LI-COR) using an iBlot2® Transfer Stack (Thermo Fisher Scientific). The membranes were then incubated with Odyssey blocking buffer (catalog #927-70001, LI-COR) for 1 hour at room temperature, followed by overnight incubation at 4 °C with rabbit monoclonal Anti-Choline Acetyltransferase (ChAT) antibody (1:1000; catalog #ZRB1012, Sigma-Aldrich). After washing three times with PBS-T for 10 minutes each, the membranes were incubated with goat anti-rabbit IgG Alexa®Fluor680 RDye (1:15,000; catalog #926-32211, LI-COR) for 1 hour at room temperature and washed with PBS three times for 10 minutes each. Western blot protein bands were captured using Odyssey CLX and analyzed with Image Studio software (LI-COR). Normalization of bands was performed using a Revert™ Total Protein (catalog #296-11021, LI-COR).

# Supplementary Figures and Tables


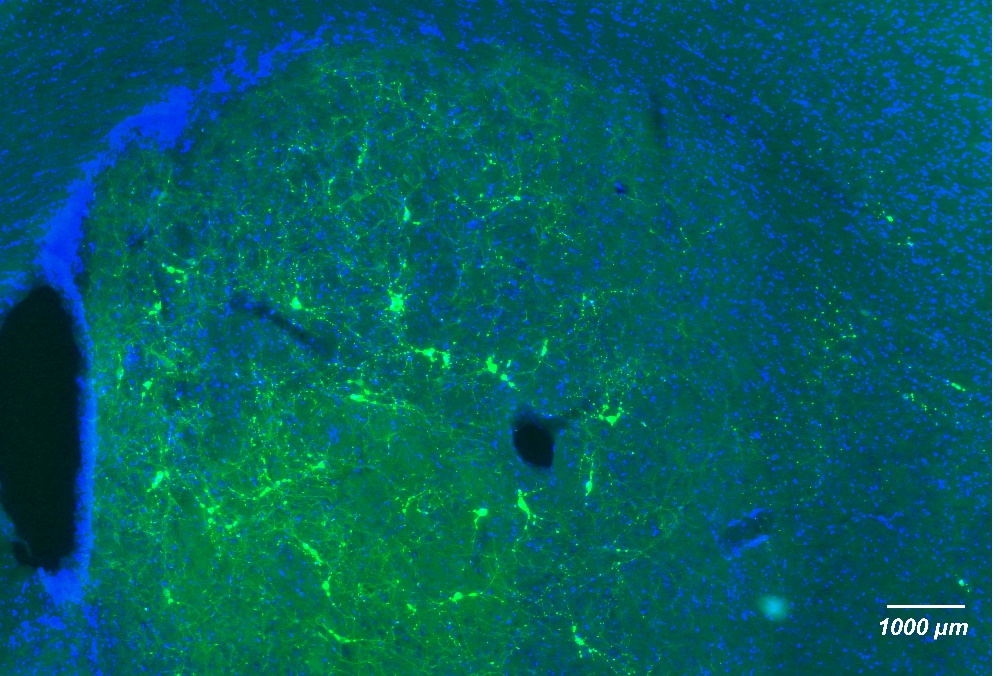


**Supplementary Figure 1. Representative image of the striatum of a knockout animal expressing Cre-dependent virus.** A. eYFP (green) and DAPI (blue). 20x magnification.


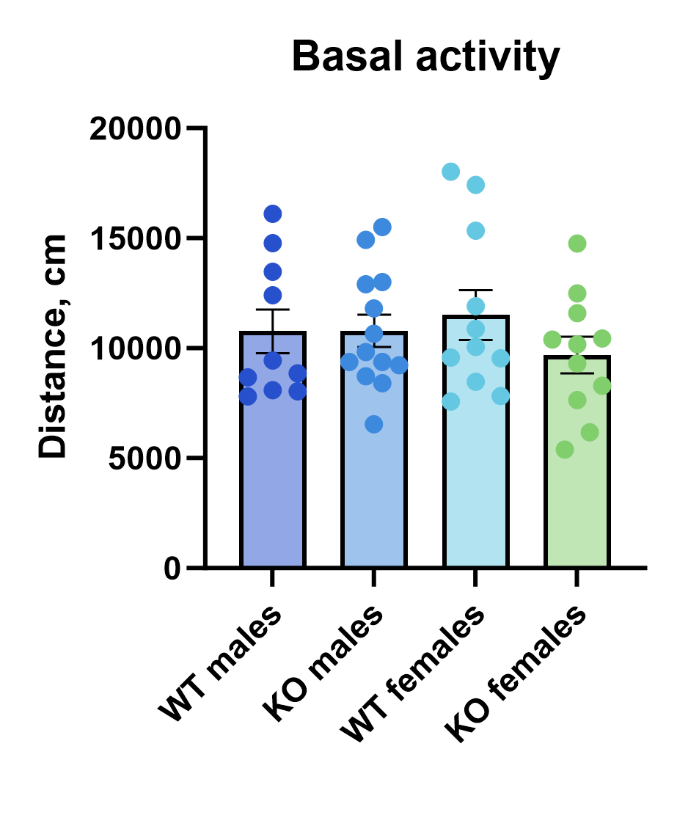


**Supplementary Figure 2. Basal locomotor activity in naïve male and female mice**.

Total distance (cm) measured in an open field for 1 hour; One-way ANOVA (p=0.58); n=10-13 per group.


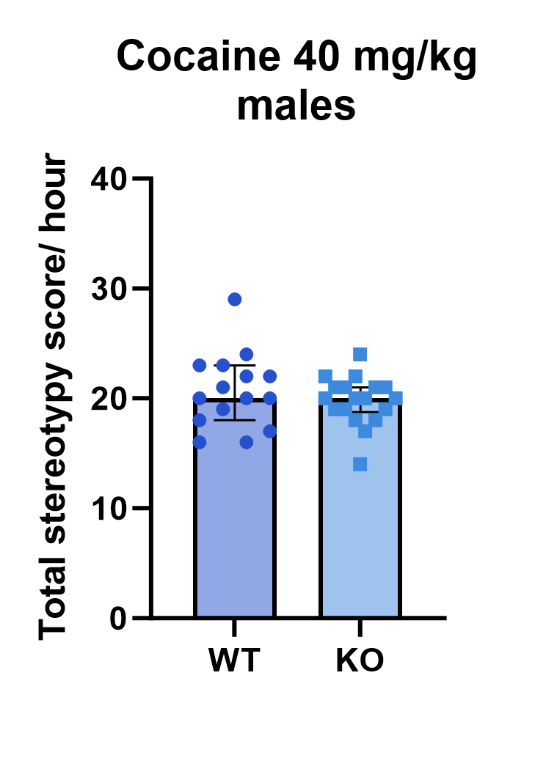


**Supplementary Figure 3**. **Cocaine-induced stereotypy in an open field.**

Abscissa: total stereotypy scoring/1 hour. Ordinate: knockout and wild type groups; The data presented as median ± IQR; n=15-18. The unpaired t-test showed no significant difference between genotypes (p=0.37).


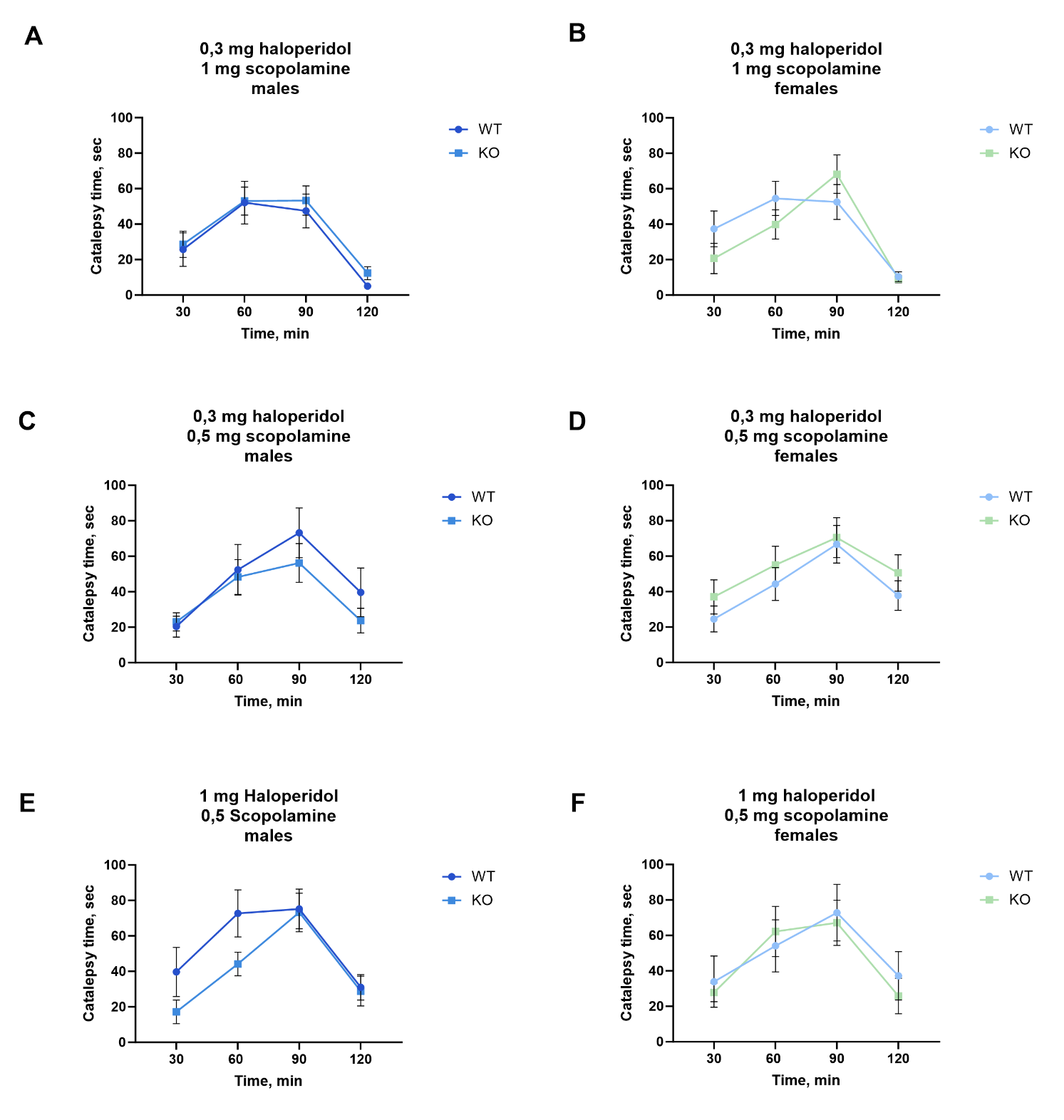


**Supplementary Figure 4. Haloperidol-induced catalepsy in male and female knockout and wild type mice.**

Catalepsy in male and female mice was measured 30, 60, and 90 min after injection of 0.3 mg/kg (A, B) or 1 mg/kg haloperidol (C, D, E, F). Haloperidol caused catalepsy in both genotypes and both sexes. Scopolamine-induced reversal was examined 120 min after haloperidol injection with 1.0 mg/kg (A, B) or 0.5 mg/kg scopolamine (C, D, E, F). Scopolamine equally reduced haloperidol-induced catalepsy in both genotypes. Abscissa: time points, measured in minutes after haloperidol injection. Ordinate: cataleptic response measured in seconds; n=11-18 per group.

**
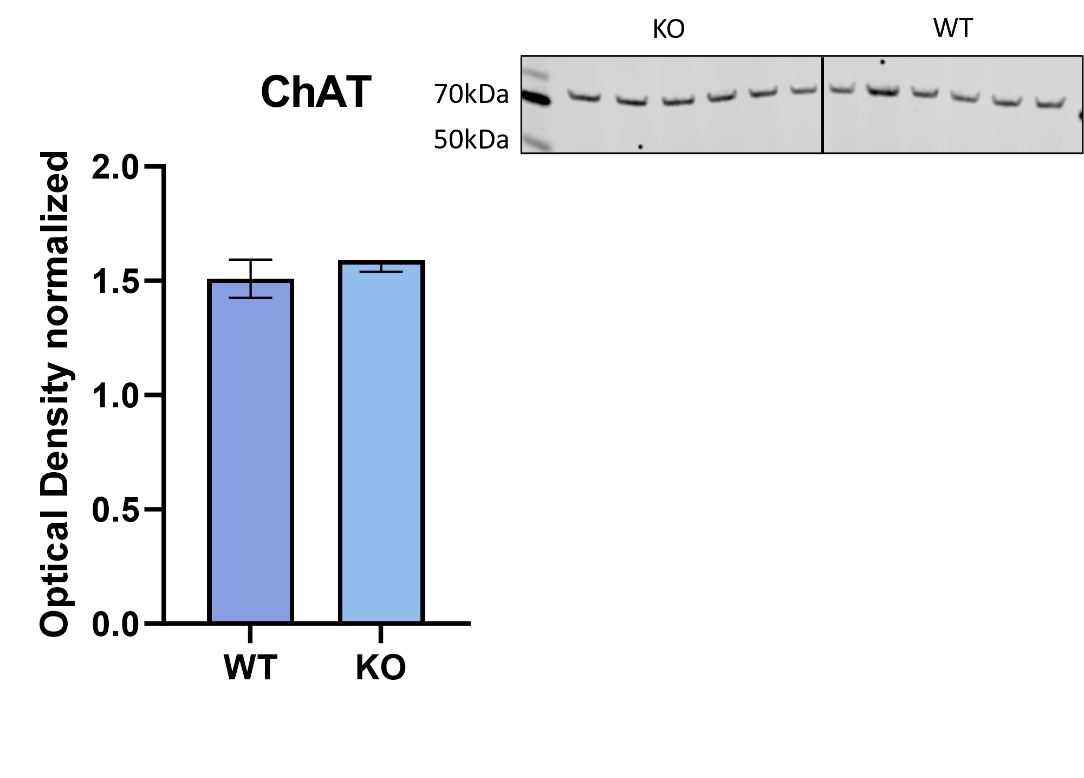
**

**Supplementary Figure 5.** **Representative immunoblot of ChAT protein.**

Representative western blot showing ChAT protein expression. The left panel shows the quantification of relative ChAT protein normalized to total stain protein. The unpaired t-test showed no significant difference between genotypes (p=0.42). The right panel shows an immunoblot of ChAT protein isolated from the striatum of knockout and wild type mice (n=6 per group) with a predicted molecular weight of ~74 kDA.


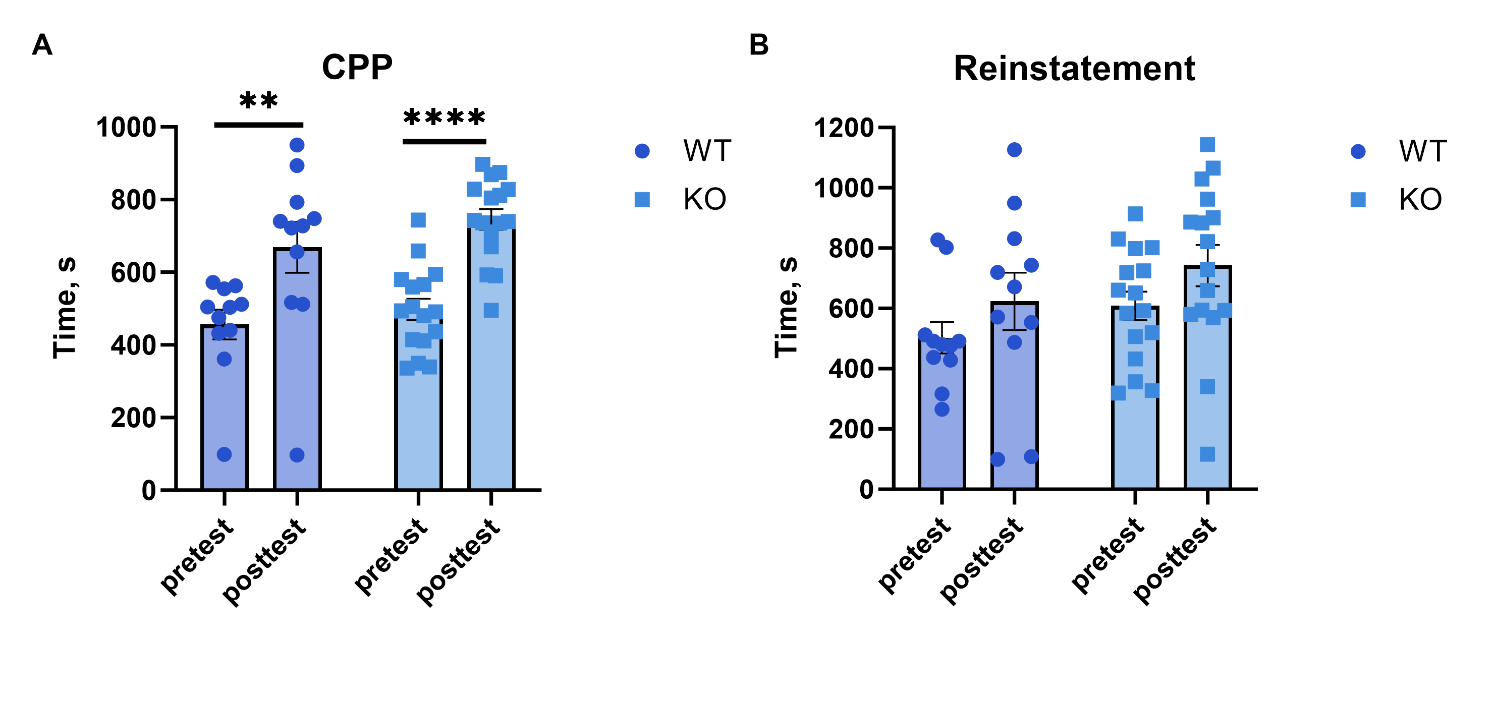


**Supplementary Figure 6. Knockout mice and wild type mice spent a similar amount of time in the cocaine-paired compartment.**

Cocaine-conditioned place preference presented as time spent in the cocaine-paired compartment on the pretest and posttest after conditioning (A), or after extinction (B). Data points represent individual animal values ± SEM. n = 11 WT, n = 16 KO. **p < 0.005, ****p < 0.0001.

**Supplementary Table 1. Assessment of basic reflexes and sensorimotor functions in M_4_-ChAT-Cre^+^ mice and their controls.**

Naïve males and females, along with their control littermates, underwent the SHIRPA behavioral screen. The data presented as means ± SEM; n=10-13, indicate that no significant differences were observed in any of the investigated measures.

| Test | M_4_-ChAT-Cre^-^ males | M_4_-ChAT-Cre^+^ males | M_4_-ChAT-Cre^-^females | M_4_-ChAT-Cre^+^ females |
| --- | --- | --- | --- | --- |
| **Viewing jar** |  |  |  |  |
| Body position  Activity  Respiration rate  Tremor | 4±0  1.9±0.1  2.0±0  0±0 | 4±0  1.8±0.1  2.0±0  0±0 | 4±0  1.8±0.1  2.0±0  0±0 | 4±0  1.5±0.1  2.0±0  0±0 |
| **Open-field** |  |  |  |  |
| Transfer arousal  Piloerection  Palpreal closure  Gait  Pelvic elevation  Tail elevation  Touch-escape (1-2)  Locomotor activity (1hour) | 4.9±0.1  0±0  0±0  0±0  2±0  1.0±0  1.0±0  10767±988 | 4.5±0.1  0±0  0±0  0±0  2±0  1.0±0  1.0±0  10793±739 | 4.8±0.1  0±0  0±0  0±0  2±0  1.1±0.1  1.0±0  11470±1250 | 4.9±0.1  0±0  0±0  0±0  2±0  1.1±0.1  1.0±0  10126±783 |
| **Tail-lifting** |  |  |  |  |
| Trunk curl  Limb grasping  Visual placing | 0±0  0±0  3.1±0.1 | 0±0  0±0  3.1±0.1 | 0±0  0±0  2.8±0.1 | 0±0  0±0  2.9±0.1 |
| **Horizontal grid** |  |  |  |  |
| Grip strength  Body tone  Pinna reflex  Corneal reflex  Toe pinch | 2.7±0.2  1.0±0  0.9±0.1  0.9±0.1  3.0±0.0 | 2.8±0.1  1.0±0  0.9±0.1  0.9±0.1  3.0±0.0 | 2.9±0.1  1.0±0  1.0±0  1.0±0  2.9±0.1 | 3.0±0  1.0±0  1.0±0  1.0±0  2.6±0.2 |
| **Horizontal wire** |  |  |  |  |
| Wire maneuver (0-1) | 2.4±0.4 | 2.8±0.3 | 2.5±0.4 | 1.6±0.5 |
| **Supine restraint** |  |  |  |  |
| Skin color  Heart rate  Limb tone  Abdominal tone  Lacrimation  Salivation  Biting | 1.0±0  1.0±0  2.1±0.2  1.0±0  0±0  0±0  0.6±0.2 | 1.1±0.1  1.0±0  1.7±0.2  1.0±0  0±0  0±0  0.5±0.1 | 1.0±0  1.0±0  1.6±0.2  1.0±0  0±0  0±0  0.8±0.2 | 1.0±0  1.0±0  1.8±0.1  1.0±0  0±0  0±0  0.4±0.2 |
| **Tube** |  |  |  |  |
| Contact righting  Vertical grid  Geotaxis | 1.0±0  0.3±0.2 | 1.0±0  0.4±0.1 | 0.9±0.1  0.4±0.2 | 0.9±0.1  0.1±0.1 |
| **Handling** |  |  |  |  |
| Fear  Irritability  Aggression  Vocalization (0-1) | 0±0  0.1±0.1  0±0  0.4±0.2 | 0±0  0.1±0.1  0±0  0.7±0.1 | 0±0  0±0  0.2±0.1  0.8±0.1 | 0±0  0±0  0±0.1  0.8±0.1 |
| **Body measures** |  |  |  |  |
| Weight (g)  Length (mm) | 25.9±0.7  79±2.0 | 26.7±1.0  80±1.0 | 20.3±0.4  81±1.0 | 20.6±0.5  78±1.0 |

**Supplementary Table 2. Details of the statistical analysis**

**Table 2a**

| **Experiment** | **Genotype Effect** | **Treatment Effect** | **Genotype x Treatment Interaction** |
| --- | --- | --- | --- |
| Cocaine-Induced Locomotor activity |  |  |  |
| - 40 mg/kg, Males | F(1, 35) = 7.808, p = 0.0084 | F(2.155, 75.44) = 12.25, p < 0.0001 | F(8, 280) = 0.214, p = 0.29 |
| - 40 mg/kg, Females | F(1, 21) = 0.2931, p = 0.59 | F(3.461, 72.68) = 7.576, p < 0.0001 | F(17, 357) = 0.65, p = 0.85 |
| Scopolamine-Induced Locomotor activity |  |  |  |
| - 1 mg/kg, Males | F(1, 22) = 0.05, p = 0.82 | F(3.251, 71.53) = 12.56, p < 0.0001 | F(5, 110) = 0.169, p = 0.97 |
| - 3 mg/kg, Males | F(1, 22) = 0.001, p = 0.97 | F(3.445, 75.79) = 50.46, p < 0.0001 | F(5, 110) = 0.3264, p = 0.89 |
| - 1 mg/kg, Females | F(1, 23) = 3.490, p = 0.07 | F(2.872, 66.06) = 47.55, p < 0.0001 | F(5, 115) = 1.582, p = 0.17 |
| - 3 mg/kg, Females | F(1, 23) = 4.384, p = 0.04 | F(3.380, 77.74) = 43.79, p < 0.0001 | F(5, 115) = 2.117, p = 0.068 |

**Table 2b**

| **Experiment** | **Genotype Effect** | **Treatment Effect** | **Day-of-Treatment Effect** | **Interaction** |
| --- | --- | --- | --- | --- |
| Cocaine Sensitization | F(1, 17) = 0.2351, p = 0.634 | F(3.806, 129.4) = 5.2, p = 0.0008 | F(3, 34) = 11.22, p < 0.0001 | F(5, 85) = 0.2351, p = 0.76 |

**Table 2c**

| **Experiment** | **Genotype Effect** | **Liquid Food Concentration Effect** |
| --- | --- | --- |
| Operant Behavior - Fixed Ratio | F(1, 124) = 1.086, p = 0.29 | F(4, 124) = 23.07, p < 0.0001 |
| Operant Behavior - Progressive Ratio | F(1, 100) = 1.933, p = 0.16 | F(4, 100) = 16.20, p < 0.0001 |

**References:**

Creese, I., & Iversen, S. D. (1973). Blockage of amphetamine induced motor stimulation and stereotypy in the adult rat following neonatal treatment with 6-hydroxydopamine. *Brain Research*, *55*(2), 369–382. https://doi.org/10.1016/0006-8993(73)90302-8

**
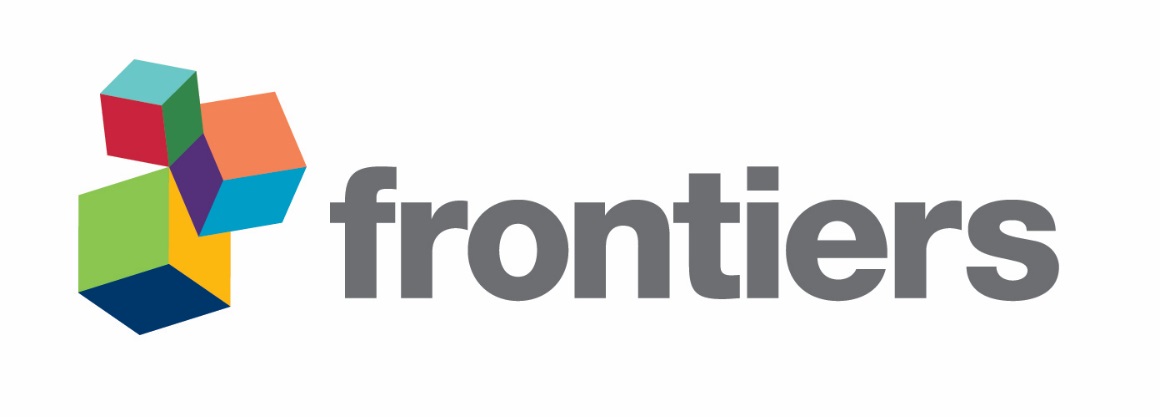
**
